# Efficacy and moderators of cognitive behavioural therapy versus interpersonal psychotherapy for adult depression: study protocol of a systematic review and individual participant data meta-analysis

**DOI:** 10.1136/bmjopen-2025-111686

**Published:** 2025-12-10

**Authors:** Tao Lin, Zachary D Cohen, Simona Stefan, Radu Șoflău, Liviu A Fodor, Raluca Georgescu, Sanne J E Bruijniks, Lotte Lemmens, Michael Bagby, Lena Quilty, Annika Ekeblad, Rolf Holmqvist, Jonathan Evans, Heather A O’Mahen, Jennifer E Johnson, Caron Zlotnick, Peter Hilpert, Janet Carter, Carolina McBride, Michael Constantino, Alice E Coyne, Holly A Swartz, Lauren M Bylsma, Eirini Karyotaki, Clara Miguel, Pandora Pantazi, Madison R Schmidt, Frederik J Wienicke, Jasmijn Breunese, Steven D Hollon, Pim Cuijpers, Ioana-Alina Cristea, Ellen Driessen

**Affiliations:** 1 Department of Psychiatry, University of Pennsylvania, Philadelphia, Pennsylvania, USA; 2 Department of Psychology, Ohio University, Athens, Ohio, USA; 3 Department of Psychology, University of Arizona, Tucson, Arizona, USA; 4 The International Institute for the Advanced Studies of Psychotherapy and Applied Mental Health, Babeș-Bolyai University of Cluj-Napoca, Cluj-Napoca, Romania; 5 Department of Clinical Psychology and Psychotherapy, Babeș-Bolyai University of Cluj-Napoca, Cluj-Napoca, Romania; 6 Department of Clinical Psychology, Utrecht University, Utrecht, The Netherlands; 7 Department of Clinical Psychological Science, Maastricht University, Maastricht, The Netherlands; 8 Department of Psychology and Psychiatry, University of Toronto, Toronto, Ontario, Canada; 9 Centre for Addiction and Mental Health, University of Toronto, Toronto, Ontario, Canada; 10 Department of Behavioral Science and Learning, Linköping University, Linkoping, Sweden; 11 University Of Bristol, Bristol, UK; 12 University of Exeter, Exeter, UK; 13 Charles Stewart Mott Department of Public Health, Michigan State University, Flint, Michigan, USA; 14 Departments of Psychiatry and Human Behavior, Brown University, Providence, Rhode Island, USA; 15 Department of Psychology, University of Lausanne, Lausanne, Vaud, Switzerland; 16 Department of Psychology, University of Canterbury, Christchurch, New Zealand; 17 Cognitive & Interpersonal Therapy Centre, Toronto, Ontario, Canada; 18 Department of Psychological and Brain Sciences, University of Massachusetts Amherst, Amherst, Massachusetts, USA; 19 Department of Psychology, American University, Washington, District of Columbia, USA; 20 Department of Psychiatry, University of Pittsburgh School of Medicine, Pittsburgh, Pennsylvania, USA; 21 Department of Clinical, Neuro and Developmental Psychology, VU University Amsterdam, Amsterdam, The Netherlands; 22 Department of Clinical Psychology, Radboud Universiteit, Nijmegen, The Netherlands; 23 Department of Clinical Psychology, Northwestern University, Evanston, Illinois, USA; 24 Department of Psychology, Vanderbilt University, Nashville, Tennessee, USA; 25 Department of General Psychology, University of Padova, Padova, Italy; 26 Depression Expertise Center, Pro Persona Mental Health Care, Nijmegen, Netherlands

**Keywords:** Depression & mood disorders, Meta-Analysis, Treatment Outcome

## Abstract

**Abstract:**

**Introduction:**

Cognitive behavioural therapy (CBT) and interpersonal psychotherapy (IPT) are both efficacious treatments for depression, but it is less clear how both compare on outcome domains other than depression and in the longer term. Moreover, it is unclear which of these two psychotherapies works better for whom. This article describes the protocol for a systematic review and individual participant data (IPD) meta-analysis that aims to compare the efficacy of CBT and IPT for adults with depression on a range of outcomes in both the short and long term, and to explore moderators of the treatment effect. This study can enhance our understanding of treatments for depression and inform treatment personalisation.

**Methods and analysis:**

Systematic literature searches will be conducted in PubMed, PsycINFO, EMBASE and the Cochrane Library from inception to 1 January 2026, to identify randomised clinical trials (RCTs) comparing CBT and IPT for adult depression. Researchers of eligible studies will be invited to contribute their participant-level data. One-stage IPD meta-analyses will be conducted with mixed-effects models to examine (a) treatment efficacy on all outcome measures that are assessed at post-treatment or follow-up in at least two studies, and (b) various baseline participant characteristics as potential moderators of depressive symptom level at treatment completion.

**Ethics and dissemination:**

Ethical approval is not required for this study since it will be based on anonymised data from RCTs that have already been completed. The findings of the present study will be disseminated through a peer-reviewed journal or conference presentation.

**Strengths and limitations of this study**

- We will examine a broad range of outcome domains in both short and longer term, which allows for a more comprehensive comparison of cognitive behavioural therapy and interpersonal psychotherapy.
- Individual participant data meta-analysis allows for examining moderators at participant level with increased statistical power compared with individual randomised clinical trials and conventional meta-analyses.
- Requesting all outcome and moderator variables assessed in the studies increases the chances of studying outcomes and moderators that might not have been reported in publications.
- Some outcomes or potential moderator variables of interest may not be examined because they are not assessed in a sufficient number of studies.
- Standardising or transforming variables that were assessed using different measures across studies can lead to loss of information from individual studies.

## Introduction

 Cognitive behavioural therapy (CBT) and interpersonal psychotherapy (IPT) are both efficacious treatments for depression, with no significant differences in efficacy emerging in recent meta-analyses of randomised clinical trials (RCTs).^1 2^ The largest network meta-analysis of psychotherapies for depression to date found no difference between the two regarding response rates, defined as 50% reduction in depressive symptoms, at post-treatment or at long-term follow-up.^1^ There were also no differences in symptom reduction or remission, the latter most often defined as absence of a major depressive disorder (MDD) diagnosis or symptom severity under a cut-off, at treatment completion. Another meta-analysis of direct comparisons included nine trials comparing CBT and IPT and reported an effect size of 0.00 (95% CI −0.12 to 0.12) for symptom severity at post-treatment.^2^ However, previous meta-analyses comparing CBT and IPT have primarily focused on post-treatment depression. Little is known regarding the relative efficacy of CBT and IPT for other outcome domains. Depression is associated with a range of mental and medical problems.^3 4^ Expanding or focusing on areas like anxiety, interpersonal functioning and well-being is essential for developing a more comprehensive understanding of how these therapies can support diverse aspects of the life of patients with depression, beyond simply addressing symptom severity.

Although CBT and IPT have comparable efficacy for depression on average, they might differ for some participant subgroups or in certain domains. For instance, a conventional meta-analysis of efficacy moderators, variables that could be associated with better response to CBT or IPT, identified one using study-level data: CBT was more effective than IPT when both were administered without concomitant antidepressant medication.^5^ Nevertheless, this study has methodological limitations, as it compared pre-post effect sizes for patients receiving CBT with pre-post effect sizes for patients receiving IPT, instead of including RCTs that directly compared CBT and IPT. Furthermore, secondary analyses of single RCTs directly comparing CBT and IPT identified some patient characteristics that could be associated with treatment response. For example, in an RCT for patients with MDD, patients’ comorbid anxiety, assessed both categorically as diagnosis and dimensionally as symptoms, was associated with better response to CBT than IPT at post-treatment but not follow-up.^6 7^ Cluster A or B personality disorder (PD) features each were associated with better response to CBT than IPT at post-treatment and follow-up.^8^ Similarly, other studies showed that CBT was marginally more effective than IPT for more severe depression and for patients with a comorbid PD.^9^,^11^ In contrast, IPT outperformed CBT in treating depression for patients with less self-sacrificing interpersonal problems.^12^

Nevertheless, if the goal is to personalise treatment by predicting which patients will respond better to which treatment, both conventional meta-analyses and single RCTs might be insufficient and potentially misleading. Conventional meta-analyses can only estimate relationships across trials, relying on trial-level characteristics or summary information for individual variables,^13^ aggregated at trial or subgroup level. This approach is subject to trial-level confounding and consequent ecological fallacy (ie, treatment covariate interactions at the aggregate level do not reflect the true interaction at individual participant level).^14^ Single trials are usually powered to detect an overall effect and thus often do not have sufficient power to detect differences among individuals.^15^ It was estimated that trial sample sizes should increase fourfold to detect genuine subgroup interaction effects.^16^ Lack of statistical power could also explain contradictory findings regarding potential treatment moderators. For example, one study reported no differences in outcomes for CBT vs IPT based on baseline severity,^7^ another found that IPT was more effective than CBT for patients with severe depression,^17^ and a third found that CBT was superior to IPT in treating severe depression.^10^ Reconciling these different moderator findings was made more difficult by inconsistencies in how ‘severe depression’ was defined across the three trials.

The significant increase in statistical power required to overcome the limitations of single RCTs in identifying moderators of treatment effect could be challenging for psychotherapy research, as it would lead to unsustainable costs, extended recruitment and prolonged trial durations. A more feasible alternative is individual participant data (IPD) meta-analysis, an approach that consists of retrieving and pooling primary data from multiple RCTs.^13^ This methodology provides a significant boost in statistical power, particularly when the various trials to be combined are of small or moderate sample size, as often is the case in psychotherapy research. It therefore allows for a more reliable investigation of treatment-covariate interactions.^13^ Moreover, definitions of outcomes and covariates can be standardised.^18^ For example, scores on different instruments can be converted to a common metric,^19 20^ aiding interpretation and clinical relevance of the results.

By incorporating IPD meta-analysis for a broader range of outcome measures (eg, anxiety, well-being) and extending our investigation into long-term effects, we aim to build on existing research and offer a more comprehensive analysis of the relative efficacy of CBT and IPT. The use of IPD meta-analysis of RCTs is pivotal to our approach, enabling a detailed examination of outcomes, time points and patient characteristics that may not be reported in published studies. Therefore, our aim is to conduct an IPD meta-analysis to examine the comparative efficacy of CBT and IPT for adults with depression on a broad range of outcome domains in the short and longer term and to examine whether individual characteristics are associated with differential treatment response.

## Method

### Design and registration

This systematic review and IPD meta-analysis will be registered in the PROSPERO International Prospective Register of Systematic Reviews after peer review of this study protocol article is completed. Additional critical amendments to the protocol will be updated in the PROSPERO register. Data extraction will start after PROSPERO registration is finalised. This study builds on prior work by our group.^21^,^24^ This protocol follows the Preferred Reporting Items for Systematic Review and Meta-Analysis Protocols (PRISMA-P) statement (see online supplemental table 1).^25^ The planned start and end dates of this study will be, respectively, November 2025 and 31 May 2026.

### Eligibility criteria

We will include randomised RCTs in which CBT alone and IPT alone are directly compared with one another for adult depression. There are no restrictions regarding the study years, publication language or publication status.^21^,^24^ We will update the searches every 4 months during the duration of the study until 1 January 2026.

To be determined as CBT, an intervention must be a manualised psychotherapy in which cognitive restructuring is the main treatment component, typically also incorporating some behavioural techniques as well, such as activity scheduling. Beck *et al*’s model is considered to be the prototype of CBT,^26^ but other CBT manuals can also be eligible for the current study.^22 24^ To be considered as IPT, an intervention must be a psychotherapy that follows Klerman & Weissman’s IPT manuals or manuals for its shorter version named interpersonal counselling.^2127^,^31^ CBT and IPT in any format (eg, individual, group) and delivery method (eg, videoconferencing, telephone, text) will be included as long as it is provided by a trained healthcare provider. Therefore, unguided self-help (eg, unguided bibliotherapy, unguided computerised interventions) will be excluded; whereas, guided self-help delivered by healthcare professionals will be included. No restriction will be placed on the treatment length, the number of therapy sessions, clinical setting (eg, outpatient, inpatient) and the duration of follow-up.^21^,^24^

Participants are required to be aged 18 years or older. Therefore, we will exclude studies including children or adolescents. Participants will be considered depressed if they meet specified criteria (eg, Diagnostic and Statistical Manual of Mental Disorders) for major depressive disorder or another unipolar mood disorder assessed by means of a semistructured interview or clinicians’ assessment, or if they present a score at or above a validated cut-off indicating the likelihood of clinically significant depressive symptoms on an evaluator-assessed, clinician-assessed or self-reported measure of depression.^21^,^24^ Participant criteria will be evaluated at study level. Consistent with previous IPD meta-analyses on depression treatments,^21^,^24^ we will exclude studies that included both eligible and non-eligible participants (eg, both children and adults).

### Outcomes and prioritisation

The primary outcome of this IPD meta-analysis will be depressive symptoms at treatment completion because we consider this as the main target of CBT and IPT for depression.^21^,^24^ The depressive symptoms must be assessed by validated self-report or clinician-rated continuous measures such as the Beck Depression Inventory (BDI)^32 33^; Hamilton Depression Rating Scale (HDRS)^34 35^; Montgomery-Asberg Depression Rating Scale^36^ or Edinburgh Postnatal Depression Scale.^37^ We will operationalise depressive symptoms as participants’ BDI-II total scores at the primary post-treatment time point as defined by the study’s authors.^24^ We chose the BDI-II as the main outcome because it is a widely used instrument that was designed to measure depression severity,^24 33^ is frequently applied in depression treatment research and is sensitive to change.^38^ We preferred the BDI-II over the HDRS,^34^ because the latter includes items that are no longer included in the modern construct of depression.^24 39^ If a study did not assess the BDI-II, we will transform the primary continuous depression measure as defined by the authors into BDI-II total scores using existing transformation schemas.^20 40^ If the primary continuous outcome depression scale administered in a study is not covered in these algorithms, we will identify the secondary (third, fourth, etc in this order) continuous depressive symptoms measure and use this for conversion. If none of the measures assessed in the study is included in conversion algorithms, we aim to develop one ourselves based on the collected IPD.^24^ We will use mean differences as the principal measure of effect for analyses with (transformed) BDI-II scores as outcome measure.

Secondary outcomes will be depressive symptoms at follow-up, defined as 6 months to 1.5 years after the treatment was completed, as well as all other outcome domains assessed at post-treatment or follow-up in at least two included studies. These domains include anxiety, general psychopathology, social/interpersonal functioning and quality of life.^21 22^ Depressive symptoms at follow-up will also be assessed with transformed BDI-II scores. As we expect the other outcome domains to be assessed with multiple instruments, we will standardise these outcomes into *z*-scores within each included study.^21 22^ We will use Cohen’s d effect sizes as the principal measures of effect for analyses with these outcomes.^21 22^ We will also report observed response rates, defined as the proportion of patients achieving ⩾50% symptom reduction from pre-treatment to post-treatment on the primary continuous depression measure.

Potential moderator variables are defined as all demographics (eg, gender, age, race and ethnicity, education, employment status, marital status), clinically relevant variables (eg, depression severity, comorbidities, age of onset of depression, previous treatments) and psychological characteristics (eg, personality traits) assessed in the study before the start of treatment.^22^,^24^ Consistent with a previous IPD meta-analysis protocol,^22^ we will examine a variable as a potential moderator if it is assessed in at least two studies. If the moderator variables were assessed using different instruments across individual studies, we will standardise continuous moderator variables into *z*-scores within each included study,^22 23^ except for baseline depression severity, for which we will use (transformed) baseline BDI-II scores.^24^ Categorical moderator variables will be recoded into similar categories.^22 23^ For raw integer variables that are meaningful and countable in nature, such as number of comorbid diagnoses, we will include the raw counts if they are uniformly defined across studies and recode them into categories if they are assessed differently across studies.

### Search methods

To identify relevant studies, we will search Metapsy (www.metapsy.org), a database of randomised clinical trials that examine the efficacy of psychological treatments for depression.^21 22 24^ The Metapsy database is developed through comprehensive literature searches in the bibliographic databases PubMed, PsycINFO, EMBASE and the Cochrane Library and is updated every 4 months. The search terms include a combination of index terms and free-text words that indicate depression and psychotherapies, with filters for randomised clinical trials (see https://osf.io/nv3ea/ and online supplemental material 1 for the exact terms used in the searches). A series of published meta-analyses have been based on the Metapsy database.^21 22 24 41^

In each new update of this database, two raters will independently screen all records based on titles, abstracts and keywords, and review relevant full-text papers to determine the eligibility for the Metapsy database, code the treatment comparison(s) examined and extract data from the included studies’ publications. Next, two raters will independently assess all full-text papers of studies marked as comparing a psychotherapy monotreatment condition against another active monotreatment condition for meeting the eligibility criteria for this work.^21 22 24^ Disagreements will be resolved through consensus at each stage. If consensus cannot be reached, a third rater will be consulted.^21^,^24^ In addition, the reference lists of included studies and reference lists of prior conventional meta-analyses that compare the efficacy of CBT and IPT for adult unipolar depression will be assessed for relevant RCTs that might have been missed.^12 5 21^,^24 42 43^

### Risk of bias assessment

Two independent raters will assess the risk of bias of the included studies related to randomisation process, deviations from intended interventions, missing outcome data, measurement of outcome and selection of the reported outcome using the Cochrane Collaboration risk of bias tool for randomised trials version 2.^24 44^ If the relevant information is not available in the publication, we will contact the authors.^21^,^24^ Disagreements will be resolved by consensus or, when consensus cannot be reached, by involving a third rater.^21^,^24^

### Data collection

We will contact authors of the included studies and invite them to participate in this project by sharing their trial’s participant-level data. Researchers who share their data for the IPD meta-analysis will be offered co-authorship on all publications stemming from the use of these data, provided that they meet internationally accepted criteria for authoring scientific articles (www.icmje.org). Furthermore, researchers who contribute data will have access to the aggregated IPD database and can use this database to investigate other research questions, on the condition of approval from the original studies’ authors for use of their data for the additional purpose. This strategy was found to be effective in convincing researchers to share their data for previous IPD meta-analyses of depression treatment.^21^,^2445 46^

We will use a multi-step contact protocol, which has been used and has proven to be successful in reaching authors of the included studies for prior depression treatment IPD meta-analyses to contact authors.^21^,^24^ Specifically, contact information of all corresponding authors will be retrieved from the publications, or if not reported or outdated through internet searches or personal contacts with other researchers. An email invitation will be sent to the corresponding authors to introduce the project’s goals and ask whether they would be willing to share their study’s IPD. If we do not receive a response from the corresponding author after 3 weeks, we will send a second and third email to follow-up. If the corresponding author remains non-responsive to these emails, we will contact the other authors via email, in this order: first, last, second, third, fourth, etc. If no response is received, a letter will be mailed to the corresponding author (again with three attempts). If these attempts fail, we will try to contact the corresponding author by telephone. If the corresponding author cannot be reached, we will contact the other authors by letter and telephone. If none of the authors respond to these efforts, we will try to use other methods to contact one of the authors (eg, via colleagues who might know them or contacting their affiliated institution). A study’s data will be considered unavailable only if (a) all of the above attempts fail or (b) an author either indicates that the IPD were not retained or declines to share these data.^21^,^24^

### Data items

We will request the following IPD items from the authors: treatment condition, all outcome variables assessed at baseline, during and after treatment (with item-level data for depression outcome measures), and all potential moderator variables assessed in the study (see online supplemental table 2 for a preliminary list of moderators). The participant-level dataset will be anonymised by the original study’s authors prior to data transfer.^21^,^24^

The following study-level characteristics will be extracted from the publications of all included studies by two independent raters: country where the study took place, recruitment method (eg, community, clinical), target group (eg, adults in general, students), depression inclusion criteria, number of CBT/IPT sessions, CBT/IPT format (eg, individual, group), CBT/IPT modality (eg, in-person, telehealth) and assessment time points.^22^ CBT/IPT treatment quality will be examined according to the following criteria: (1) whether a treatment manual was used, (2) whether the therapy was provided by trained therapists and (3) whether treatment integrity was verified.^22^ Additionally, for the purpose of meta-bias assessment, effect size data for all outcomes will be extracted from the publications for studies for which IPD are and are not available. If the publication does not report relevant information on study-level characteristics or treatment quality, or effect size data, we will request it from the authors.^21 22 24^

### Data integrity checks

Consistent with previous IPD meta-analyses for depression treatment,^21^,^24^ we will conduct three data integrity checks after receiving the dataset. First, we will check whether the dataset includes the full intention-to-treat sample (ie, all participants randomised to treatment) and otherwise matches what is reported in the published article. To this end, we will calculate baseline characteristics as well as observed mean scores of outcomes at pre-treatment and post-treatment using the dataset and compare them to the scores reported in the article. Second, we will check whether any outcome and potential moderator variables reported in the article are missing in the dataset. Third, we will check outcome and moderator variables for inconsistent, invalid or out-of-range items. If any discrepancies arise during these data integrity checks, we will discuss them with the authors.^21^,^24^

### IPD meta-analyses

To facilitate comparison of this study’s findings to previous IPD meta-analyses on depression treatment efficacy, we will adopt the data-analysis strategy applied in the prior studies.^21^,^23^ More details of this strategy can be found in prior publications.^21^,^23^ This strategy is based on the approach recommended by Twisk *et al*^47^ and is chosen because it adequately accounts for baseline values and effectively addresses missing data. Specifically, it allows participants with only baseline data but missing assessments at post-treatment and/or follow-up to remain included in the analyses without requiring multiple imputation.^48^

We will perform one-stage IPD meta-analyses on intention-to-treat samples, as far as data are available, using three-level mixed-effects models (level 1: measurement moment; level 2: participant; level 3: study) with restricted maximum likelihood estimation.^21^,^23^ As some participants may seek additional help during the follow-up period, which usually cannot be controlled, follow-up data will be excluded from post-treatment analyses. To examine heterogeneity, the variance among studies as a proportion of the total variance (ie, I^2^-statistic) will be calculated by dividing the random effect variance for study by the sum of all variance components. Furthermore, the normality assumption will be checked by visually inspecting histograms of (standardised) residuals.^22^ To compare the efficacy of CBT and IPT, separate models will be estimated for each outcome variable. These models will include a main effect for time and a time×treatment interaction.^47^ Time will be included as a dummy variable in order to compare treatments at the different time points. Additionally, the model will estimate a random intercept with respect to study to account for the clustering of participants within studies and a random intercept with respect to participants to control for the clustering of repeated measures within participants.^21 22 49^ The models will contain random slopes for time and time×treatment interaction on the study level. The regression coefficient of the time×treatment interaction will be considered to reflect the treatment effect.^21 22 49^

To examine moderator effects, we will estimate separate models for each potential moderator variable. These models will add a main effect for the moderator, a time×moderator interaction, and a time × treatment × moderator three-way interaction to the model described above.^22^ The models will also contain random slopes on the study level of the main effect for the moderator, time×moderator interaction, and time × treatment × moderator three-way interaction on the study level. In case of convergence issues, the random effect structure will be simplified. A significant three-way interaction is considered to indicate a moderator effect. Finally, we will model all significant moderators simultaneously to assess their independence. For categorical moderator variables, separate treatment effects will be estimated for each moderator category. For significant continuous moderator variables, the interaction will be probed with simple slope analysis. For each outcome and potential moderator, the strength of the body of evidence will be assessed based on the number of included studies and participants, as well as the quality of the included studies.^21 22^ P values will be calculated with Kenward-Roger’s approximate F test and 95% CIs will be calculated using bootstrapping with 10 000 simulations. The analyses will be performed in R with the lme4 package.^50^

### Sensitivity analyses

We will conduct seven analyses to examine potential study-level moderators of CBT vs IPT efficacy concerning depressive symptoms at post-treatment. First, to examine the robustness of the findings to those eligible for CBT and IPT for depression in general mental healthcare, we will repeat the analysis for studies including general adult populations meeting diagnostic criteria for a mood disorder and providing in-person CBT and IPT, excluding studies in which guided self-help is provided.^22^ Second, to examine whether the risk of bias of the included studies impacts outcome, we will repeat the analysis in studies with low risk of bias.^21 22^ This approach allows us to examine whether the inclusion of studies with some or serious concerns about bias impacts the overall findings. Third, we will examine the impact of CBT and IPT quality by repeating the analysis in studies that score high treatment quality on all criteria.^22^ Fourth, to examine the impact of the number of IPT sessions, we will perform the analyses separately in the subgroup of studies examining IPT and the subgroup of studies examining its briefer version (ie, interpersonal counselling). Fifth, to investigate the potential differences between Beckian CBT and other CBT approaches, we will repeat the analysis in the subgroups of studies that cite the Beck treatment manual and the subgroup of studies that used other treatment manuals.^22 26^ Sixth, to examine the impact of depression type, we will perform the analyses separately in the subgroup of studies focusing on MDD and the subgroup of studies on postpartum depression. Finally, we will conduct a sensitivity analysis adding the variable(s) used for stratified randomisation in the primary studies as covariate(s) to the model.

### Meta-biases

Following the recommendations proposed by Sterne and colleagues,^51^ we will assess potential publication bias or small study effects by examining asymmetry in contour-enhanced funnel plots with Egger’s test for the intercept in the meta-analyses including 10 or more studies.^21 22^ These funnel plots will include both studies for which IPD are and are not available and will be based on the effect size data extracted from the publications.^21 22^

We will examine potential data availability bias by comparing characteristics between studies for which IPD are and are not obtained with t-tests for continuous variables and χ^2^-tests analyses for categorical variables.^21^,^23^ Additionally, we will conduct conventional meta-analysis ‘subgroup analyses’—applying a fully random-effects analysis and pooling study-to-study variance across subgroups—to compare effect sizes between studies for which IPD were and were not available. For these analyses, Cohen’s d effect sizes will be calculated based on the effect size data extracted from the publications.

### Ethics and dissemination

Institutional review board (IRB) approval is not required for this IPD meta-analysis, since it will be based on anonymised data from RCTs that have already been completed.^21^,^24^ Depending on their institution’s policies, however, authors may be required to obtain IRB approval to share their IPD. In this case, the authors are responsible for obtaining IRB approval. By signing the data-sharing agreement, researchers will declare that (a) these IPD were collected and are shared in accordance with all applicable local and international laws and regulations and (b) all IPD will be anonymised, and no personal data will be transferred.^21^,^24^ The findings of the present study will be disseminated through a peer-reviewed journal publication and conference presentations.

### Patient and public involvement

Patients and/or the public were not involved in the design, conduct, or reporting or dissemination plans of this research.

## Discussion

This article described the protocol for a systematic review and IPD meta-analysis examining the comparative efficacy and moderators of CBT and IPT for adult depression. By identifying RCTs that compare the two psychotherapy approaches and collecting their IPD for meta-analyses, we aim to compare the efficacy of CBT and IPT on an array of outcomes at post-treatment and follow-up, and to examine which treatment works best for whom.

### Strengths and limitations

This IPD meta-analysis has several strengths. First, while previous conventional meta-analyses have narrowly focused on post-treatment depression, we will examine a broad range of outcome domains in both short and longer term,^21 22^ which allows for a more comprehensive comparison of the two psychotherapy approaches. Second, requesting all outcome and all potential moderator variables assessed in the studies increases the chances of accessing data that might not have been reported in publications and will facilitate the examination of a range of outcome domains and potential moderators.^21^,^24^ Using intention-to-treat samples as far as available and applying the same data analysis strategy across all studies, this IPD meta-analysis can further improve the reliability and precision of the effect estimates compared with prior conventional meta-analyses.^21 22^ Third, IPD meta-analysis allows for examining moderators at participant level with increased statistical power compared with individual RCTs and conventional meta-analyses.^21^,^23^

Several limitations of the proposed study should be noted. First, although IPD meta-analyses can produce more reliable and less biased results compared with conventional meta-analyses,^52^ they are not bias-free.^21^ We will reduce potential selection bias by performing a systematic literature search that poses no restrictions concerning publication language, date and status. We will empirically assess publication bias and examine data availability bias by comparing study characteristics between studies for which IPD are and are not available. Second, because IPD meta-analyses are based on completed trials, it is likely that some outcome or potential moderator variables of interest cannot be examined because they are not assessed in a sufficient number of studies.^21^,^23^ Meta-analyses of different outcomes or moderators might also be based on different subgroups of studies. In addition, constructs will likely be assessed using different measures across studies. Thus, it is necessary to standardise or transform variables to include all relevant studies. For categorical variables that were assessed with different categories or levels across studies, we will need to recode them into the broadest common category, which can lead to loss of information from individual studies that had more specific or additional categories.^21 22^ Furthermore, the moderator findings of this study will be observational in nature. Prospective validation of significant moderators in new RCTs will be needed before applying them to inform treatment personalisation.^21 22^ Finally, in real-world settings, patients might not have the chance to choose between IPT and CBT (as well as other evidence-based psychotherapies) due to limited resources and constrained treatment accessibility. While our findings will provide valuable insights into the comparative efficacy of these treatments, their effectiveness and applicability will depend on healthcare access and policy considerations.

### Clinical and scientific relevance

CBT and IPT are both effective treatments recommended in current practice guidelines for depression and are widely adopted in clinical practice.^1 53 54^ It is therefore of great importance to understand how they compare in terms of efficacy. This proposed work will contribute to the literature by providing more precise effect size estimates than prior conventional meta-analyses and extend the scope to outcome measures other than depression in both short and longer term.

Furthermore, previous RCTs have suggested that the efficacy of CBT and IPT might differ for different patient subgroups,^5^,^712^ highlighting the importance of treatment personalisation. However, the findings were often inconsistent and potentially misleading,^7 10 17^ as single RCTs were often underpowered for moderation analyses. This IPD meta-analysis will allow for the examination of moderators at the individual participant level with increased statistical power. To the best of our knowledge, this will be the first IPD meta-analysis that examines moderators across clinical trials assessing the efficacy of CBT and IPT. As such, performing this IPD meta-analysis will be an important next step towards personalising treatment selection for depression.

## Supplementary material

10.1136/bmjopen-2025-111686 online supplemental file 1
