# Supplementary material for: Efficacy and moderators of cognitive behavioural therapy versus interpersonal psychotherapy for adult depression: study protocol of a systematic review and individual participant data meta-analysis
Source: BMJ Open. 2025 Dec 10;15(12):e111686. doi: 10.1136/bmjopen-2025-111686 (PMC12699710; doi:10.1136/bmjopen-2025-111686)
Supplement: online supplemental file 1 [file bmjopen-15-12-s001.docx]

**Supplemental Table 1**

PRISMA-P (Preferred Reporting Items for Systematic review and Meta-Analysis Protocols) 2015 checklist: recommended items to address in a systematic review protocol*

| **Section and topic** | **Item No** | **Checklist item** | **Reported on page** |
| --- | --- | --- | --- |
| **ADMINISTRATIVE INFORMATION** | | |  |
| Title: |  |  |  |
| Identification | 1a | Identify the report as a protocol of a systematic review | 1 |
| Update | 1b | If the protocol is for an update of a previous systematic review, identify as such | NA |
| Registration | 2 | If registered, provide the name of the registry (such as PROSPERO) and registration number | 10 |
| Authors: |  |  |  |
| Contact | 3a | Provide name, institutional affiliation, e-mail address of all protocol authors; provide physical mailing address of corresponding author | 1 |
| Contributions | 3b | Describe contributions of protocol authors and identify the guarantor of the review | 31 |
| Amendments | 4 | If the protocol represents an amendment of a previously completed or published protocol, identify as such and list changes; otherwise, state plan for documenting important protocol amendments | 10 |
| Support: |  |  |  |
| Sources | 5a | Indicate sources of financial or other support for the review | 31 |
| Sponsor | 5b | Provide name for the review funder and/or sponsor | 31 |
| Role of sponsor or funder | 5c | Describe roles of funder(s), sponsor(s), and/or institution(s), if any, in developing the protocol | 31 |
| **INTRODUCTION** | | |  |
| Rationale | 6 | Describe the rationale for the review in the context of what is already known | 7 |
| Objectives | 7 | Provide an explicit statement of the question(s) the review will address with reference to participants, interventions, comparators, and outcomes (PICO) | 9 |
| **METHODS** | | |  |
| Eligibility criteria | 8 | Specify the study characteristics (such as PICO, study design, setting, time frame) and report characteristics (such as years considered, language, publication status) to be used as criteria for eligibility for the review | 10 |
| Information sources | 9 | Describe all intended information sources (such as electronic databases, contact with study authors, trial registers or other grey literature sources) with planned dates of coverage | 13 |
| Search strategy | 10 | Present draft of search strategy to be used for at least one electronic database, including planned limits, such that it could be repeated | 13 |
| Study records: |  |  |  |
| Data management | 11a | Describe the mechanism(s) that will be used to manage records and data throughout the review | 14 |
| Selection process | 11b | State the process that will be used for selecting studies (such as two independent reviewers) through each phase of the review (that is, screening, eligibility and inclusion in meta-analysis) | 13 |
| Data collection process | 11c | Describe planned method of extracting data from reports (such as piloting forms, done independently, in duplicate), any processes for obtaining and confirming data from investigators | 13 |
| Data items | 12 | List and define all variables for which data will be sought (such as PICO items, funding sources), any pre-planned data assumptions and simplifications | 15 |
| Outcomes and prioritization | 13 | List and define all outcomes for which data will be sought, including prioritization of main and additional outcomes, with rationale | 11 |
| Risk of bias in individual studies | 14 | Describe anticipated methods for assessing risk of bias of individual studies, including whether this will be done at the outcome or study level, or both; state how this information will be used in data synthesis | 14 |
| Data synthesis | 15a | Describe criteria under which study data will be quantitatively synthesised | 16 |
|  | 15b | If data are appropriate for quantitative synthesis, describe planned summary measures, methods of handling data and methods of combining data from studies, including any planned exploration of consistency (such as I^2^, Kendall’s τ) | 16 |
|  | 15c | Describe any proposed additional analyses (such as sensitivity or subgroup analyses, meta-regression) | 18 |
|  | 15d | If quantitative synthesis is not appropriate, describe the type of summary planned | NA |
| Meta-bias(es) | 16 | Specify any planned assessment of meta-bias(es) (such as publication bias across studies, selective reporting within studies) | 20 |
| Confidence in cumulative evidence | 17 | Describe how the strength of the body of evidence will be assessed (such as GRADE) | 18 |

**Supplemental Table 2**

Potential Moderators in IPD Meta-analysis

| Category | Variables |
| --- | --- |
| Patient characteristics | Age |
|  | Gender |
|  | Marital status |
|  | Race |
|  | Ethnicity |
|  | Education level |
|  | Employment status |
|  | Income |
|  | Living situation |
|  | Children status |
|  | Religion |
|  | Current psychosocial problem |
|  | Physical exercise |
|  | BMI |
| Depression characteristics | Depression type |
|  | Current depression episode |
|  | Current episode duration |
|  | Depression severity |
|  | Number of previous depression episodes |
|  | Suicide risk (ideation, attempts) |
| Clinical history | Previous therapy |
|  | Previous hospitalization |
|  | Age of depression onset |
|  | Previous psychiatric disorder |
|  | Age of first psychiatric disorder onset |
|  | Comorbidity (e.g., anxiety, bipolar, personality disorder) |
|  | Childhood trauma |
|  | Alcohol use |
|  | Smoking status |
|  | Use of antidepressants |
| Psychological patient characteristics | Personality trait |
|  | Attachment style |
|  | Coping style |
|  | Treatment expectations |

**Supplementary Material 1**

Search strings for four bibliographical databases.

The search strategies were preregistered on <https://osf.io/nv3ea>.

Cuijpers, P., Karyotaki, E., Ciharova, M., Quero, S., Pineda, B., Munoz, R., Struijs, S. Y., Llamas, J., Figueroa, C., Marinho Vieira, P., & Rosenström, T. (2021, February 23). *A meta-analytic database of randomised trials on psychotherapies for depression* [OSF project]. Open Science Framework. <https://doi.org/10.17605/OSF.IO/825C6>

**PubMed**

Psychotherapy [MH] OR psychotherap*[All Fields] OR cbt[All Fields] OR "behavior therapies"[All Fields] OR "behavior therapy"[All Fields] OR "behavior therapeutic"[All Fields] OR "behavior therapeutical"[All Fields] OR "behavior therapeutics"[All Fields] OR "behavior therapeutist"[all Fields] OR "behavior therapeutists"[All Fields] OR "behavior treatment"[All Fields] OR "behavior treatments"[All Fields] OR "behaviors therapies"[All Fields] OR "behaviors therapy"[All Fields] OR "behaviors therapeutics"[All Fields] OR "behaviors therapeutic"[All Fields] OR "behaviors therapeutical"[All Fields] OR "behaviors therapeutist"[All Fields] OR "behaviors therapeutists"[All Fields] OR "behaviors treatment"[All Fields] OR "behaviors treatments"[All Fields] OR "behavioral therapies"[All Fields] OR "behavioral therapy"[All Fields] OR "behavioral therapeutics"[All Fields] OR "behavioral therapeutic"[All Fields] OR "behavioral therapeutical"[All Fields] OR "behavioral therapeutist"[All Fields] OR "behavioral therapeutists"[All Fields] OR "behavioral treatment"[All Fields] OR "behavioral treatments"[All Fields] OR "behaviour therapies"[All Fields] OR "behaviour therapy"[All Fields] OR "behaviour therapeutic"[All Fields] OR "behaviour therapeutical"[All Fields] OR "behaviour therapeutics"[All Fields] OR "behaviour therapeutist"[all Fields] OR "behaviour therapeutists"[All Fields] OR "behaviour treatment"[All Fields] OR "behaviour treatments"[All Fields] OR "behaviours therapies"[All Fields] OR "behaviours therapy"[All Fields] OR "behaviours therapeutics"[All Fields] OR "behaviours therapeutic"[All Fields] OR "behaviours therapeutical"[All Fields] OR "behaviours therapeutist"[All Fields] OR "behaviours therapeutists"[All Fields] OR "behaviours treatment"[All Fields] OR "behaviours treatments"[All Fields] OR "behavioural therapies"[All Fields] OR "behavioural therapy"[All Fields] OR "behavioural therapeutics"[All Fields] OR "behavioural therapeutic"[All Fields] OR "behavioural therapeutical"[All Fields] OR "behavioural therapeutist"[All Fields] OR "behavioural therapeutists"[All Fields] OR "behavioural treatment"[All Fields] OR "behavioural treatments"[All Fields] OR "cognition therapies"[All Fields] OR "cognition therapie"[All Fields] OR "cognition therapy"[All Fields] OR "cognition therapeutical"[All Fields] OR "cognition therapeutic"[All Fields] OR "cognition therapeutics"[All Fields] OR "cognition therapeutist"[All Fields] OR "cognition therapeutists"[All Fields] OR "cognition treatment"[All Fields] OR "cognition treatments"[All Fields] OR psychodynamic[All Fields] OR Psychoanalysis[MH] OR psychoanalysis[All Fields] OR psychoanalytic*[All Fields] OR counselling[All Fields] OR counseling[All Fields] OR Counseling[MH] OR "problem-solving"[All Fields] OR mindfulness[All Fields] OR (acceptance[All Fields] AND commitment[All Fields] ) OR "assertiveness training"[All Fields] OR "behavior activation"[All Fields] OR "behaviors activation"[All Fields] OR "behavioral activation"[All Fields] OR "cognitive therapies"[All Fields] OR "cognitive therapy"[All Fields] OR "cognitive therapeutic"[All Fields] OR "cognitive therapeutics"[All Fields] OR "cognitive therapeutical"[All Fields] OR "cognitive therapeutist"[All Fields] OR "cognitive therapeutists"[All Fields] OR "cognitive treatment"[All Fields] OR "cognitive treatments"[All Fields] OR "cognitive restructuring"[All Fields] OR (("compassion-focused"[All Fields] OR "compassion-focussed"[All Fields]) AND (therapy[SH] OR therapies[All Fields] OR therapy[All Fields] OR therape*[All Fields] OR therapis*[All Fields]OR Therapeutics [OR treatment*[All Fields])) OR ((therapy[SH] OR therapies[All Fields]

OR therapy [All Fields] OR therape*[All Fields] OR therapis*[All Fields] OR Therapeutics[MH] OR treatment*[All Fields]) AND constructivist*[All Fields]) OR "metacognitive therapies"[All Fields] OR "metacognitive therapy"[All Fields] OR "metacognitive therapeutic"[All Fields] OR "metacognitive therapeutics"[All Fields] OR "metacognitive therapeutical"[All Fields] OR "metacognitive therapeutist"[All Fields] OR "metacognitive therapeutists"[All Fields] OR "metacognitive treatment"[All Fields] OR "metacognitive treatments"[All Fields] OR "meta-cognitive therapies"[All Fields] OR "meta-cognitive therapy"[All Fields] OR "meta-cognitive therapeutic"[All Fields] OR "meta-cognitive therapeutics"[All Fields] OR "meta-cognitive therapeutical"[All Fields] OR "meta-cognitive therapeutist"[All Fields] OR "meta-cognitive therapeutists"[All Fields] OR "meta-cognitive treatment"[All Fields] OR "meta-cognitive treatments"[All Fields] OR "solution-focused therapies"[All Fields] OR "solution-focused therapy"[All Fields] OR "solution-focused therapeutic"[All Fields] OR "solution-focused therapeutics"[All Fields] OR "solution-focused therapeutical"[All Fields] OR "solution focused therapies"[All Fields] OR "solution focused therapy"[All Fields] OR "solution focused therapeutic"[All Fields] OR "solution focused therapeutics"[All Fields] OR "solution focused therapeutical"[All Fields]OR "solution-focussed therapies"[All Fields] OR "solution-focussed therapy"[All Fields] OR "solution-focussed therapeutic"[All Fields] OR "solution-focussed therapeutics"[All Fields] OR "solution-focussed therapeutical"[All Fields]OR "solution focussed therapies"[All Fields] OR "solution focussed therapy"[All Fields] OR "solution focussed therapeutic"[All Fields] OR "solution focussed therapeutics"[All Fields] OR "solution focussed therapeutical"[All Fields] OR "self-control therapies"[All Fields] OR "self-control therapy"[All Fields] OR "self-control therapeutics"[All Fields] OR "self-control therapeutical"[All Fields] OR "self-control therapeutic"[All Fields] OR "self-control training"[All Fields] OR "self-control trainings"[All Fields] OR "self control therapies"[All Fields] OR "self control therapy"[All Fields] OR "self control therapeutics"[All Fields] OR "self control therapeutical"[All Fields] OR "self control therapeutic"[All Fields] OR "self control training"[All Fields] OR "self control trainings"[All Fields]

AND

(Depressive Disorder[MH] OR Depression[MH]OR dysthymi*[All Fields] OR "affective disorder"[All Fields]OR "affective disorders"[All Fields] OR "mood disorder"[All Fields] OR "mood disorders"[All Fields] OR depression*[All Fields] OR depressive*[All Fields] OR "dysthymic disorder"[MeSH Terms])

Limits: RCTs

**Embase**

#1

'psychotherapy'/exp OR 'psychotherapy' OR 'psychotherapies' OR 'psychotherapeutics' OR 'psychotherapeutical' OR 'cognitive therapy'/exp OR 'cognitive behavior therapy'/exp OR 'behavior therapy'/exp OR ´cbt´ OR ´cognitive behavioural therapy´ OR ´cognitive behavioural therapies´ OR cognitive behavioral therapy´ OR 'cognitive behavioral therapies' OR 'behavior therapy' OR 'behavior therapies' OR 'behaviour therapy' OR 'behaviour therapies' OR 'cognition therapy' OR 'cognitive therapies' OR 'cognitive therapy' OR 'cognitive therapeutic' OR 'cognitive therapeutics' OR 'cognitive therapeutical' OR 'cognitive therapeutist' OR 'cognitive therapeutists' OR 'cognitive treatment' OR 'cognitive treatments' OR 'cognitive restructuring' OR 'cognition therapies' OR 'cognition therapie' OR 'cognition therapeutical' OR 'cognition therapeutic' OR 'cognition therapeutics' OR 'cognition therapeutist' OR 'cognition therapeutists' OR 'cognition treatment' OR 'cognition treatments' OR 'behavior therapeutic' OR 'behavior therapeutical' OR 'behavior therapeutics' OR 'behavior therapeutist' OR 'behavior therapeutists' OR 'behavior treatment' OR 'behavior treatments' OR 'behaviors therapies' OR 'behaviors therapy' OR 'behaviors therapeutics' OR 'behaviors therapeutic' OR 'behaviors therapeutical' OR 'behaviors therapeutist' OR 'behaviors therapeutists' OR 'behaviors treatment' OR 'behaviors treatments' OR 'behavioral therapies' OR 'behavioral therapy' OR 'behavioral therapeutics' OR 'behavioral therapeutic' OR 'behavioral therapeutical' OR 'behavioral therapeutist' OR 'behavioral therapeutists' OR 'behavioral treatment' OR 'behavioral treatments' OR 'behaviour therapeutic' OR 'behaviour therapeutical' OR 'behaviour therapeutics' OR 'behaviour therapeutist' OR 'behaviour therapeutists' OR 'behaviour treatment' OR 'behaviour treatments' OR 'behaviours therapies' OR 'behaviours therapy' OR 'behaviours therapeutics' OR 'behaviours therapeutic' OR 'behaviours therapeutical' OR 'behaviours therapeutist' OR 'behaviours therapeutists' OR 'behaviours treatment' OR 'behaviours treatments' OR 'behavioural therapies' OR 'behavioural therapy' OR 'behavioural therapeutics' OR 'behavioural therapeutic' OR 'behavioural therapeutical' OR 'behavioural therapeutist' OR 'behavioural therapeutists' OR 'behavioural treatment' OR 'behavioural treatments' OR 'behavior activation' OR 'behaviors activation' OR 'behavioral activation' OR 'behaviour activation' OR 'behaviours activation' OR 'behavioural activation' OR 'psychoanalytic therapy'/exp OR 'psychodynamic' OR 'psychodynamical' OR 'psychoanalysis' OR 'psychoanalytical' OR 'counselling'/exp OR 'counseling'/exp OR 'counselling' OR 'counseling' OR 'problem-solving' OR 'problem solving' OR 'supportive therapy' OR 'metacognitive therapy' OR 'metacognitive therapies' OR 'metacognitive therapeutic' OR 'metacognitive therapeutics' OR 'metacognitive therapeutical' OR 'metacognitive therapeutist' OR 'metacognitive therapeutists' OR 'metacognitive treatment' OR 'metacognitive treatments' OR 'meta-cognitive therapy' OR 'meta-cognitive therapies' OR 'meta-cognitive therapeutic' OR 'meta-cognitive therapeutics' OR 'meta-cognitive therapeutical' OR 'meta-cognitive therapeutist' OR 'meta-cognitive therapeutists' OR 'meta-cognitive treatment' OR 'meta-cognitive treatments' OR 'solution-focused therapies' OR 'solution focused therapies' OR 'solution-focussed therapies' OR 'solution focused therapies' OR 'solution-focused therapy' OR 'solution focused therapy' OR 'solution-focussed therapy' OR 'solution focused therapy' OR 'solution-focused therapeutic' OR 'solution focused therapeutic' OR 'solution-focussed therapeutic' OR 'solution focussed therapeutic' OR 'solution-focused therapeutics' OR 'solution focused therapeutics' OR 'solution-focussed therapeutics' OR 'solution focused therapeutics' OR 'solution-focused therapeutical' OR 'solution focused therapeutical' OR 'solution-focussed therapeutical' OR 'solution focused therapeutical' OR 'self-control therapies' OR 'self control therapies' OR 'self-control therapy' OR 'self control therapy' OR 'self-control therapeutics' OR 'self control therapeutics' OR 'self-control therapeutical' OR 'self control therapeutical' OR 'self-control therapeutic' OR 'self control therapeutic' OR 'self-control training' OR 'self control training' OR 'self control trainings' OR 'self-control trainings' OR 'mindfulness' OR 'acceptance commitment' OR 'acceptance and commitment' OR 'assertiveness training'

#2

'compassion-focused' OR 'compassion-focussed' OR 'compassion focused' OR 'compassion focussed' OR 'constructivist' OR 'constructivists'

#3

'therapies' OR 'therapy' OR 'therapeutics' OR 'therapist' OR 'treatment' OR 'treatments'

#4

Combine: #2 AND #3

#5: #1 OR #4

#6

'depressive disorder'/exp OR 'depression'/exp OR 'depressive' OR 'major depression'/exp OR 'major depressive disorder'/exp OR 'depression' OR 'depressions' OR 'depressive' OR 'dysthymic disorder'/exp OR 'dysthymic disorder' OR 'dysthymia'/exp OR 'dysthymic' OR 'mood disorder'/exp OR 'affective disorder'/exp OR 'affective disorder' OR 'affective disorders' OR 'mood disorder' OR 'mood disorders'

Combine: #5 AND #6

Limits: RCTs

**PsycINFO**

(DE "Psychotherapy" OR "Psychotherapy" OR "psychotherapies" OR "psychotherapeutic" OR "psychotherapeutical" OR "psychotherapeutics" OR DE "Behavior Therapy" OR DE "Cognitive Behavior Therapy" OR "CBT" OR "behavior therapies" OR "behavior therapy" OR "behavior therapeutic" OR "behavior therapeutical" OR "behavior therapeutics" OR "behavior therapeutist" OR "behavior therapeutists" OR "behavior treatment" OR "behavior treatments" OR "behaviors therapies" OR "behaviors therapy" OR "behaviors therapeutics" OR "behaviors therapeutic" OR "behaviors therapeutical" OR "behaviors therapeutist" OR "behaviors therapeutists" OR "behaviors treatment" OR "behaviors treatments" OR "behavioral therapies" OR "behavioral therapy" OR "behavioral therapeutics" OR "behavioral therapeutic" OR "behavioral therapeutical" OR "behavioral therapeutist" OR "behavioral therapeutists" OR "behavioral treatment" OR "behavioral treatments" OR "behaviour therapies" OR "behaviour therapy" OR "behaviour therapeutic" OR "behaviour therapeutical" OR "behaviour therapeutics" OR "behaviour therapeutist" OR "behaviour therapeutists" OR "behaviour treatment" OR "behaviour treatments" OR "behaviours therapies" OR "behaviours therapy" OR "behaviours therapeutics" OR "behaviours therapeutic" OR "behaviours therapeutical" OR "behaviours therapeutist" OR "behaviours therapeutists" OR "behaviours treatment" OR "behaviours treatments" OR "behavioural therapies" OR "behavioural therapy" OR "behavioural therapeutics" OR "behavioural therapeutic" OR "behavioural therapeutical" OR "behavioural therapeutist" OR "behavioural therapeutists" OR "behavioural treatment" OR "behavioural treatments" OR "cognition therapies" OR "cognition therapie" OR "cognition therapy" OR "cognition therapeutical" OR "cognition therapeutic" OR "cognition therapeutics" OR "cognition therapeutist" OR "cognition therapeutists" OR "cognition treatment" OR "cognition treatments" OR "cognitive therapies" OR "cognitive therapy" OR "cognitive therapeutic" OR "cognitive therapeutics" OR "cognitive therapeutical" OR "cognitive therapeutist" OR "cognitive therapeutists" OR "cognitive treatment" OR "cognitive treatments" OR "cognitive restructuring" OR DE "Emotion Focused Therapy" OR DE "Psychoanalysis" OR "psychoanalysis" OR "psychoanalytic" OR "psychoanalytical "OR DE "Psychodynamic Psychotherapy" OR "psychodynamic" OR DE "Psychotherapeutic Counseling" OR "counselling" OR "counseling" OR "problem-solving" OR "problem solving" OR "mindfulness" OR ("acceptance" AND "commitment") OR "assertiveness training" OR "behavior activation" OR "behaviors activation" OR "behavioral activation" OR "behaviour activation" OR "behaviours activation" OR "behavioural activation" OR "metacognitive therapies" OR "metacognitive therapy" OR "metacognitive therapeutic" OR "metacognitive therapeutics" OR "metacognitive therapeutical" OR "metacognitive therapeutist" OR "metacognitive therapeutists" OR "metacognitive treatment" OR "metacognitive treatments" OR "meta-cognitive therapies" OR "meta-cognitive therapy" OR "meta-cognitive therapeutic" OR "meta-cognitive therapeutics" OR "meta-cognitive therapeutical" OR "meta-cognitive therapeutist" OR "meta-cognitive therapeutists" OR "meta-cognitive treatment" OR "meta-cognitive treatments" OR DE "Solution Focused Therapy" OR "solution-focused therapies" OR "solution-focused therapy" OR "solution-focused therapeutic" OR "solution-focused therapeutics" OR "solution-focused therapeutical" OR "solution-focussed therapies" OR "solution-focussed therapy" OR "solution-focussed therapeutic" OR "solution-focussed therapeutics" OR "solution-focussed therapeutical" OR "solution focused therapies" OR "solution focused therapy" OR "solution focused therapeutic" OR "solution focused therapeutics" OR "solution focused therapeutical" OR "solution focussed therapies" OR "solution focussed therapy" OR "solution focussed therapeutic" OR "solution focussed therapeutics" OR "solution focussed therapeutical" OR "self-control therapies" OR "self-control therapy" OR "self-control therapeutics" OR "self-control therapeutical" OR "self-control therapeutic" OR "self-control training" OR "self-control trainings" OR "self control therapies" OR "self control therapy" OR "self control therapeutics" OR "self control therapeutical" OR "self control therapeutic" OR "self control training" OR "self control trainings" OR (("compassion-focused" OR "compassion-focussed" OR "compassion focused" OR "compassion focussed") AND ("therapies" OR "therapy" OR "therapie" OR "therapist" OR "therapists" OR "therapeut" OR "treatment" OR "treatments")) OR ("constructivist" AND ("therapies" OR "therapy" OR "therapie" OR "therapist" OR "therapists" OR "therapeut" OR "treatment" OR "treatments")))

AND

(DE "Depression (Emotion)" "depressive disorder" OR "depression" OR "depressions" OR "depressive" OR DE "Major Depression" OR "major depression" OR "major depressive disorder" OR DE "Dysthymic Disorder" OR "Dysthymia" OR " dysthymic disorder" OR DE "Affective Disorders" OR "Affective Disorder" OR "affective disorders" OR "Mood Disorder" OR "Mood disorders")

Limits: Methodology is ME=(treatment outcome/clinical trial): papers (December 2014)

**Cochrane**

#1 MeSH descriptor: [Depressive Disorder] explode all trees : 6, 777

#2 ''depress*'' (Word variations have been searched) : 51, 768

#3 #1 or #2 : 51, 783

#4 ''major depressive disorder'' (Word variations have been searched) : 5, 435

#5 #3 or #4 : 51, 783

#6 MeSH descriptor: [Dysthymic Disorder] explode all trees : 129

#7 ''dysthymi*'' (Word variations have been searched) : 649

#8 #6 or #7 : 649

#9 #5 or #8 : 51, 800

#10 ''mood disorder'' (Word variations have been searched) :4, 034

#11 ''affective disorder'' (Word variations have been searched) : 2, 882

#12 #10 or #11 : 6, 055

#13 #9 or #12 : 53, 227

#14 MeSH descriptor: [Psychotherapy] explode all trees : 13, 568

#15 ''psychotherap*'' (Word variations have been searched) : 7, 758

#16 ''CBT'' (Word variations have been searched) : 2, 029

#17 ''Cognitive Behav* therap* (Word variations have been searched) : 8, 893

#18 #14 or #15 or #16 or #17 : 20, 795

#19 'psychodynamic'' (Word variations have been searched) : 469

#20 MeSH descriptor: [Psychoanalysis] explode all trees : 13

#21 ''psychoanaly*'' (Word variations have been searched) : 345

#22 MeSH descriptor: [Counseling] explode all trees : 2, 783

#23 ''counseling*'' (Word variations have been searched) : 6, 913

#24 ''problem solving'' (Word variations have been searched) : 2, 867

#25 #18 or #19 or #20 or #21 or #22 or #23 or #24 : 28, 149

#26 ''acceptance commitment'' (Word variations have been searched) : 168

#27 ''assertiveness training'' (Word variations have been searched) :231

#28 ''behavior activation'' (Word variations have been searched) : 663

#29 ''mindfulness'' (Word variations have been searched) : 466

#30 ''metacognitive therap*'' (Word variations have been searched) :56

#31 ''solution focused therap*'' (Word variations have been searched) :858

#32 ''self control training'' (Word variations have been searched): 5850

#33 #25 or #26 or #27 or #28 or #29 or #30 or #31 or #32 : 32, 748

#34 ''Randomized Controlled Trial'':ti,ab,kw (Word variations have been searched) : 120, 901

#35 #13 and #33 and #34 in Trials: 4,614

Limit: publication year: 1970-2018
