# Supplementary material for: Efficacy and moderators of cognitive behavioural therapy versus interpersonal psychotherapy for adult depression: study protocol of a systematic review and individual participant data meta-analysis
Source: BMJ Open. 2025 Dec 10;15(12):e111686. doi: 10.1136/bmjopen-2025-111686 (PMC12699710; doi:10.1136/bmjopen-2025-111686)
Supplement: Supplementary file 2 [file bmjopen-2025-111686.reviewer_comments.pdf]

PEER REVIEW HISTORY

BMJ Open publishes all reviews undertaken for accepted manuscripts. Reviewers are asked to complete a checklist review form and are provided with free text boxes to elaborate on their assessment. These free text comments are reproduced below.

ARTICLE DETAILS

Title (Provisional)

Efficacy and moderators of cognitive behavioral therapy versus interpersonal psychotherapy for adult depression: study protocol of a systematic review and individual participant data meta-analysis

Authors

Lin, Tao; Cohen, Zachary D.; Stefan, Simona; Șoflău, Radu; Fodor, Liviu A; Georgescu, Raluca; Bruijniks, Sanne J. E.; Lemmens, Lotte; Bagby, Michael; Quilty, Lena; Ekeblad, Annika; Holmqvist, Rolf; Evans, Jonathan; O'Mahen, Heather A; Johnson, Jennifer E.; Zlotnick, Caron; Hilpert, Peter; Carter, Janet; McBride, Carolina; Constantino, Michael; Coyne, Alice E.; Swartz, Holly A; Bylsma, Lauren M.; Karyotaki, Eirini; Miguel, Clara; Pantazi, Pandora; Schmidt, Madison R.; Wienicke, Frederik J.; Breunese, Jasmijn; Hollon, Steven D; Cuijpers, Pim; Cristea, Ioana-Alina; Driessen, Ellen

---

VERSION 1 - REVIEW

**Reviewer**	**1**
**Name**	**Vladimir Trkulja,**
**Affiliation**	**University of Zagreb School of Medicine**
**Date**	**14-Jan-2025**
**COI**	**None**

This protocol describes a study intended to deal with an important question, and was submitted by a research group with a top-level expertise in the field (CBT, IPT) and the proposed methodology (IPD meta-analysis). I have only a few comments that the authors might wish to consider.

1. The protocol depicts a research intended to provide a comprehensive insight into the relative efficacy of CBT vs. IPT in major depression, with a particular emphasis on potential effect moderators – as an effort of identifying patient subsets in which one or the other technique would be preferable (the list of the patient-level moderators to be explored is provided in the Supplement – by the way, it seems that the word “onset” is misspelled in the Supplement as “onsite”). The authors intend to include RCTs in which CBT-IPT were applied without concomitant antidepressants. Hence, antidepressant use is not among the potential effect modifiers. In the Introduction, the authors provide a brief overview of previous meta-analyses that intended to detect moderators of the CBT vs. IPT relative effect. Among many, one previous meta-analysis is mentioned (as ref. 5) that, apparently, indicated that regarding the depression symptom severity at the end of treatment, CBT was superior to IPT but only in patients without concominant antidepressants. This would imply that the authors of the present protocol considered it not needed to again address antidepressant use as

a moderator (since, apparently, this question might be considered resolved). However, the cited meta-analysis (ref. 5) is – in my view – methodologically problematic in that the conclusion about superiority of CBT over IPT in patients without pharmacological treatments was not based on RCTs with direct CBT vs. IPT comparisons. Rather, the authors considered trials with CBT and estimated post- vs. pre- differences, and trials with IPT (and post- vs. pre- differences). For both, the estimated pooled effects (Hedges g) were high (around 1.5), but apparently somewhat higher for CBT than for IPT (resulting in a "statistically significant difference", i.e., P-value of 0.037 for the difference between these post- vs. pre- differences). I sincerely doubt that the cited meta-analysis should be viewed as an indicator of the moderating effect of "concurrent antidepressants", and particularly not as evidence to support superiority of CBT over IPT in "non-antidepressant patients". Hence, as the proposed protocol intends to be the most comprehensive one to date, it seems plausible that this potential moderator is considered, since both CBT and IPT are commonly used together with antidepressants. Some other moderators (e.g., personality disorder) were also addressed in previous meta-analyses (as indicated by the Protocol authors), yet, personality traits are one of the proposed (potential) moderators. Hence, I do not see why a potential moderator, highly likely to be seen in daily practice, is omitted from the planed analysis.

2. One of the proposed sensitivity analyses is an analysis limited to studies with "low risk of bias". The authors state "*Second, to examine whether the risk of bias of the included studies impacts outcome, we will repeat the analysis in studies with low risk of bias*." I am aware that it is a common practice to conduct meta-analyses across the strata of "study quality". However, it seems to me that this practice is not very logical: i) if the objective of a meta-analysis is to generate the most (at the moment) precise estimate possible; and ii) the most unbiased one – why would anyone include "serious concerns" or "some concerns" RCTs (as an overall judgment, e.g., in line with the RoB-2 tool) into a meta-analysis? I mean, "serious concerns" means that the trial estimate is highly likely biased (whether towards or away from the null); i.e., it means that the trial was likely estimating bias, not the treatment effect. Why would one want to include an estimate of bias into the estimate of the treatment effect? Another logical fallacy – at least in my opinion – is the view that by showing, for example, "numerically similar estimates in low- and high quality RCTs", one documents "robustness of the findings": i) the high quality and low quality trials estimate two different things, and their "overall pooled estimate" is meaningless; ii) numerical similarity of estimates in low quality and high quality trials does not mean that "trial quality does not change the treatment effect estimate", because in the former, this is not what is estimated. We do not know what would have been the treatment effect estimate in low-quality RCTs – had they been of high quality. Not to mention limitations of the quality scores (e.g., Greenland&O'Rourke Biometrics 2001; 2:463). Hence, in any meta-analysis intended to indeed provide an unbiased and precise estimate of a causal treatment effect RCTs that are viewed as problematic in terms of various biases, should most likely be omitted. Alternatively, the main analysis could be based only on RCTs deemed to be fairly unbiased, whereas supplemental data could address those RCTs that are judged as likely biased to some relevant extent. Interpretation of possible similarity or differences between two estimates is not straightforward. Eventually, one could explore effects across different domains that have been considered as sources of bias. For example, estimate in trials with low risk of treatment allocation bias and those with a high risk of such a bias – to indicate, potentially, how much this type of bias skewes the estimates, and so on. A number of other sensitivity analyses are planned. Most of those, however, induce some

questions/considerations: i) one sensitivity analysis aims to include only the RCTs with in-person delivered treatments (guided self-help are excluded). Depicting this as a "sensitivity analysis" implies that all the moderators would be evaluated not only in the entire dataset, but also specifically in those with in-person therapies. Are in-person and guided self-help the same intervention? If they are, why would one expect a moderating effect of some patient-level characteristic to differ between the in-patient RCTs and self-help RCTs; or, if the two are the same intervention – why should one consider the analysis in only in-person RCTs as a "sensitivity analysis"? On the other hand, if they are not – why would one want to generate a pooled estimate across different types of treatments: in-person CBT vs in-person IPT and self- help CBT vs self-help IPT? Would it not be more plausible to first consider the "delivery type" as a trial-level moderator? And, if it turns-out to indeed be a moderator of the CBT vs. IPT difference, then test person-level moderators in these two subsets defined by the "delivery type" which renders CBT and IPT different treatments (ie., in-person CBT is different from guided self- help CBT, in-person IPT is different from guided self-help IPT). At least to me, these are somewhat unclear points; ii) one sensitivity analysis is intended to include only RCTs with a high treatment quality on all criteria. Does this mean that here in-person treatment delivery and self- help delivery would be again analyzed jointly? What if it turns out that moderating effects of person-level characteristics are different in "delivery type" subsets…is it not counterintutive to then consider them jointly again in this analysis? iii) the fourth and the fifth sensitivity analyses were considered in (a) trials with IPT and trials with "briefer IPT counseling"; and (b) trials implementing Beckian CBT and trials with other CBT procedures. This is a bit confusing: if one criterion is "type of IPT", then either one could be compared to the Beckian CBT and "other CBT". These seem to be rather heterogeneous subsets. Let's assume that for example "age" is a person- level moderator of the Beckian CBT vs. briefer IPT difference, but not of any other difference (e.g., Beckian CBT vs. IPT, non-Beckian CBT vs. briefer IPT, or non-Beckian CBT vs. IPT). Is it not possible that then a true moderating effect would not be seen, even if it existed, because it would be "diluted" by the lack of the moderating effect in all other comparisons? Overall, all these mentioned points listed in the section "Sensitivity analyses" are a bit confusing, at least to me as a half-lay person. It seems to me that all these listed planned analyses denoted as "sensitivity analyses" actually better fit into the evaluation of study-level moderators. I am not aware of the total number of existing primary RCTs comparing CBT to IPT, but I assume that the number is likely considerable (otherwise, by simple logic, it would not be plausible for the authors to plan the proposed research). This enables the meta-regression approach. The protocol never mentions the term "meta-regression" (on individual patient data); but, in general, the depicted models including treatment*patient-level moderator interaction terms are meta-regression models, and could also include treatment*study level moderator interaction terms. Moreover, patient-level and study-level covariate interactions could be evaluated (although this may not be required for achieving the main purpose of the planned study). Also, it might be plausible to consider trials in post-partum depression separately from those in major depressive disorder.

As the sixth "sensitivity analysis" authors state: "*Finally, to adequately account for baseline values of the outcome measure and assist in the comparison of this study's findings to prior work, we will repeat the primary outcome efficacy analysis using the strategy based on the approach recommended by Twisk*

and colleagues, which has been utilized in previous depression treatment IPD meta-analyses." I belive that baseline depression score-adjusted analysis of the end-of- treatment depression scores has to be the main analysis, not the sensitivity analysis. As depicted in the Protocol, the primary outcome is "end-of-treatment" Beck II depression score (as measured or as transformed from other depression-quantifying instruments). Such an outcome has to be analyzed with adjustment for the baseline depression severity (Twisk et al. Contemp Clin Trials Commun 2018; 10:80-85; Fitzmaurice GM, Laird NM, Ware JH. Longitudinal data analysis. Wiley 2004). Next, the authors state that for the purpose of adjusting for baseline, the "method described by Twisk" is to be used. In another part of the Protocol, they denote the analysis model as a multilevel model with two random intercepts (patient; study), and fixed effects treatment, time, treatmet*time interaction. This introduces some confusion. The "Twisk model" actually means that for longitudinal continuous outcome, baseline value of the outcome is treated as an outcome, not a covariate (ie., outcome value at time 0), and this approach adjusts for the baseline value when "treatment" is not in the model, but treatment*time interaction is in the model. However, the primary outcome is a single-time point outcome – at the end of treatment, i.e., there is only one post-baseline value. Hence, the model might be a simple model without repeated measures: hence, there are fixed effects – treatment, and baseline disease severity; and random intercept for "trial" (subjects clustered in trials) (although, some argue that the more appropriate approach is to consider trials as strata – e.g., Riley, Tierney, Stewart. Individual participant data meta-analysis, Wiley 2021 – Chapter 6 on one-step procedure – however, I cannot judge the appropriatness of one or the other approach- statistical expertise is needed). Another model could include also treatment*covariate interaction. The model could of course also explore the random slope for any of the tested covariates, or for the treatment*covariate interaction (when testing the possible moderation). The question is - what would be the purpose of including also random slopes, except to improve the model fit/reduce residual heterogeneity. This might need to be clearly stated (although, for the analysis of the primary outcome, I do not really see an explicit need for such an outcome). Hence, there seems to be some confusion about the explicit mention of the "method described by Twisk" for the primary outcome analysis and the way in which the model is described. However, an expert in IPD should evaluate this point. But, whatever method is used – the primary analysis of the primary outcome should include adjustment for the baseline depression score. In fact, all analyses of all outcomes should include adjustment for the baseline value of the respective trait. Additional argument in this regard: different trials will differ with respect to the baseline scores; I assume that the CBT vs IPT trials are generally not too large, so quite some imbalances in baseline severity between CBT and IPT (in all aspects of psychopathology) should be expected in many of the primary trials.

3. It is also likely that in many primary RCTs there would be imbalances in a number of other patient-level characteristics – potential confounders. The authors plan to generate an overall estimate of the treatment effect (ie., CBT vs. IPT difference), among other goals. This estimate, I believe, should be generated not only with an account for the baseline disease severity, but likely with adjustment for other relevant covariates (selected based on the authors' subject knowledge). I assume that not many primary RCTs (if any at all) have applied stratified randomization – but if any has implemented it, then I believe that the model should include stratifying factors as covariates. Overall – it seems reasonable to assume that most of the primary studies are limited in size, thus limited in their ability to detect and adjust for imbablances in relevant confounders, and that most have not reported effect estimates adjusted for the baseline severity. IPD meta-analysis is an opportunity to rectify these shortcomings of the primary studies.

4. As mentioned, it is reasonable to assume that most of the primary studies are comparably limited in size. However, it is reasonable to assume that the really small ones (e.g. with 10-20 patients per treatment arm) would indicate larger treatment effects (particularly if published) than the somewhat large ones (e.g., 50-60-80 patients per treatment arm). The protocol anticipates the funnel plot analysis, but it seems to me that the trial size is a reasonable study- level characteristic that could be considered as an effect moderator. An overall estimate based on the combination of overestimates (from very small trials), and estimates that are likely to be closer to the truth (ie.,

from reasonably larger trials) might not be too informative. If there are indications of the "effect inflation" (small trial effect), the really informative estimate of the CBT vs. IPT differences should probably come exclusively from somewhat larger trials.

5. It is planned to test a rather large number of potential patient-level moderators. The rationale is clear, and this is a valuable effort – in the sense of contribution to the informed choices for individual patients. Still, strictly speaking, the proposed separate models (for the primary outcome) for individual potential moderators – as much as they might be informative – are also quite limited in their reach. Let's assume that age, baseline disease severity, and educational status are found to be effect modifiers, each in a separate analysis. This cannot provide an answer about a preferred choice for a middle-aged, moderately depressed and highly educated patient. Of course, no single trial or IPD MA could answer that, hence the authors rightfully depict the moderator exploration as a hypothesis-generating effort – findings of which should be evaluated in future RCTs. Still, a question remains- what other adjustments should be included in the moderator-testing models? In my opinion, if the essential condition for a reasonabley accurate estimate is to adjust for the baseline depression score – then it should be included in all models evaluating effect modifiers.

6. The Protocol envisages that eventually a model would be fitted to the primary outcome with all "significant moderators" as indepedents to evaluate their independence. The authors might want to consider the following: i) will the model also include (by default) the baseline depression score? I believe that it should; ii) Let's assume that e.g., 4 patient-level effect modifiers are detected – what fixed effects will the model include: treatment, baseline depression, each of the 4 effect modifiers, and interaction terms of each of them with treatment? Or?; iii) As outlined, it could be understood that the model would actually evaluate effects of these variables on the outcome, but this does not seem reasonable, i.e., what would be the purpose? It is true that, commonly, the effect modifiers are also confounders, i.e., that they are both associated with the outcome (or "affect the outcome") and also modify the effect of the exposure of primary interest (in this case - form of psychotherapy) – but, an effect modifier needs not be also a confounder (see e.g. VanderWeele TJ. Explanation in causal inference, Oxford University Press 2015). Assume, for example, that one of the effect modifiers is found out not to be "independently associated with the outcome" – does this disqualify it from being an effect modifier? What is gained by such an analysis?; iv) Is the criterion of "significant modifier" (which, presumably, would be based on "statistical significance of the interaction term") the appropriate one? I mean, it could be that not all trials provide data on a particular effect modifier, hence the sample size is reduced, and power is reduced, and the "test fails" in the sense that the p-value is, say, P=0.200 – should it be "disqualified" although by all reasonable arguments, or as shown in some previous study, it is likely to indeed be an effect modifier; v) Should all potential confounders be indiscriminately included? Let's assume that the history of a previous treatment (yes/no) is associated with or "causes" (at least in part) the level of depression at the trial baseline, and that it affects the outcome by affecting the baseline disease severity, which is a factor that qualifies a patient for the trial – that is, that baseline disease severity is a mediator of the effect of "previous treatment" – by adjusting for both, one would actually "block" the effect of "previous treatment" on the outcome: does one really need to condition on both?

7. Several analyses are envisaged to evaluate meta-biases. Among others, the authors intend to perform also a two-stage IPD – estimates from RCTs with available IPD will be combined with summary data from other RCTs for which IPD are not available. I have no idea about the expected number of IPD-available and non-available studies, but I presume that the latter might be even more numerous than the former. The authors plan to pool individual IPD estimates and summary estimates by the random-effects method. This appears as a reasonable effort in order to improve the precision

of the estimates. However, I am not certain that this would contribute to the accuracy of the estimates. The effect estimates based on IPD could (and should) be adjusted for the baseline scores, or potentially other covariates – resulting, likely, in improved “unbiasedness”. It seems somewhat counterintuitive to merge them with summary-based estimates likely generated from RCTs without adjustment for (at least) baseline scores. Assuming that there is a considerable number of IPD-available RCTs, the precision of the estimates would likely be considerable. It might not be the best choice to “taint” them with summary data estimates for the purpose of improving already satisfactory precision. The authors might consider a possibility of analyzing “summary data RCTs” separately, using for example bivariate meta-regression (availabe in metafor, based on van Houwelingen, Arends, Stijnen. Stat Med 2002; 21:589) – it could generate average outcome scores adjusted for average baseline scores by treatment. Then, in the next step, the overall single-stage IPD estimate and the summary data adjusted estimate might be presented to illustrate consistency (or, possible inconsistency) of the estimates.

8. The authors state the use of the method “described by Twisk” (Twisk et al. 2018) to analyze longitudinal data (e.g., data on outcome values at the end of treatment and later follow-ups), using an argument that this enables inclusion also of patients with only baseline scores, but missing later or follow-up data. This is a bit confusing. In the cited reference by Twisk and colleagues, 4 methods are described to analyze repeated measures data in RCTs, two of which are considered inadequate, since they do not account for the baseline values; and two that do. One of them, is the “longitudinal data analysis” – baseline value is considered as outcome, not covariate, and if the model does not include the factor “treatment” but only treatment*time interaction, then it provides adjustment of the post-baseline scores for the baseline value. An equally viable alternative is to use baseline as a covariate. But, inclusion of patients with no post- baseline values is meaningless – unless one implements some form of data imputation. Clearly, most of the IPD-available trials will have patients with missing data on the primary outcome (end-of-study depression score), or at later time-points, or missing data on other outcomes – is data imputation envisaged? There is no mention in the protocol, but the statement that the analysis will be on the ITT principles implies that it will. The type of data imputation needs to be declared. LOCF? Sensitivity analysis might actually be an analysis of data with a different type of data imputation (e.g., some of the multiple imputation methods).

9. Clearly, if any method indicates lower end-of-treatment Beck II depression score, this would indicate “less depression” with the respective treatment. But this does not necessarily mean that either method actually relieved depression – it could simply mean that with one vs. the other, patients were “less worse” than at the start of treatment. Of course, this is not likely, since both CBT and IPT are known to relieve depression. Still, one analysis intended to define MCID for Beck II based on data from 3 RCTs in patients with depression (Button et al. Psychol Med 2015; 40:3269), indicated, for example, average reductions in Beck II scores of -7 to -8 score points in patients who on the Global impression-improvement reported “feeling the same”. With a large number of data in IPD, a difference between, e.g, by 7.5 vs. by 6.8 reduced scores (hence, assuming closely similar baseline – by 0.7 score difference in end-of-treatment score) could be found “statistically significant” – but it would be at the same time clinically meaningless. Moreover, the MCID with Beck II was found to depend on the baseline severity. In my opinion, to make the propsed analyses fully meaningful and practically relevant: i) all outcome score analyses need to be adjusted for the baseline value; ii) it would be informative to include another outcome (as it is done in e.g., regulatory RCTs of antidepressants) – proportion of people yielding at least 50% reduction in Beck

II scores at the end of treatment. Alternatively, the outcome could be proportion of people experiencing MCID in Beck II score (which, to my understanding, could be approximated at around 25% reduction; Button et al. 2015; Kounali et al. Psychol Med 2022; 52:1875; Bdi Beck Depression Inventory Scoring - Consensus Academic Search Engine).

**Reviewer**	**2**
**Name**	**Anne Doherty**
**Affiliation**	**Galway University Hospitals, National University of Ireland - Galway**
**Date**	**14-Jan-2025**
**COI**	**None**

This is a well-written protocol which outlines a planned, systematic review in detail. The rationale and the background literature are well described. The design is thoughtful in than rather than performing a meta-analysis of study outcomes, it will seek the original trial data in order to perform analysis at an individual level. The methods are appropriate to the stated aims and are described in detail.

**Reviewer**	**3**
**Name**	**Tanya Hauck,**
**Affiliation**	**Centre for Addiction and Mental Health**
**Date**	**14-Jan-2025**
**COI**	**None**

Overall this is a very detailed and thoughtful protocol.
The authors will use participant level data from randomised clinical trials to compare IPT and CBT for depression. The goal of the study is to conduct an individual participant data meta-analysis to understand treatment efficacy and how baseline characteristics moderate outcomes.
It is well described and detailed, and the methods are reasonable.
I have a few minor points to consider, and understand that it may now be possible to address all of them, also due to data limitations:
(a) Another important domain of baseline characteristics may be therapist factors. For example, therapist training, therapist profession/role (psychologist, psychiatrist, social worker, peers, etc.), and the use of rating scales such as CTRS to rate whether the therapy is being performed properly. At least in my jurisdiction it is vastly easier to obtain training in CBT compared to IPT, and therapist familiarity with the model may also drive outcomes.
(b) IPT is formally meant to focus on four general areas (grief, conflict, changes in relationships/life, social isolation) and it is possible someone may be appropriate for CBT who does not technically qualify for IPT. If it is possible to see this eligibility criteria in the data, it would help to include it.
(c) On page 17 (page 10) it states that cognitive restructuring defines CBT. I would argue that behavioural activation is equally effective and part of the wider CBT framework, and is in the CANMAT 2016 guidelines (2016, Vol. 61(9) 524-539, Can J Psych) as equivalent to CBT for acute phase depression. I am not sure why BA is excluded? It may be particularly effective in some populations. If there are clear reasons for excluding BA, please detail them if possible.
(d) Finally, it may be worth adding to the limitations that in real practice, most jurisdictions (in public or insured systems, versus private-pay), the vast majority of clients worldwide (even in wealthy nations) will never have the option of selecting IPT versus CBT, and that resource limitations and availability of any evidenced based psychotherapy is a major concern.

**Reviewer**	**4**
**Name**	**Hisashi Noma,**
**Affiliation**	**The Institute of Statistical Mathematics, The Graduate University for Advanced Studies**
**Date**	**14-Jan-2025**
**COI**	**None**

This paper provides a protocol for an individual participant data meta-analysis that compares the efficacy of cognitive behavioral therapy and interpersonal psychotherapy. This study also aimed to explore their effect moderators. The research methods are well considered and well presented. I'd like to provide several minor comments and suggestions.

1. For calculating I^2-statistics, how are they defined in the one-stage analyses by mixed-effects models?
2. P.17, line 35: "The models will contain random and fixed slopes for time, treatment, and time × treatment interaction on the study level."
Does this statement mean all of these coefficients are random? Or, some of them are not random? In the statistical analysis section, they should be explicitly notified. Ishak et al. (2007) will be helpful for the modelling strategies.
3. P.17, line 38: "The regression coefficient of the time × treatment interaction will be considered to reflect the treatment effect."
The coefficients of interaction terms would be difficult to be interpreted. The least-squares means can be easily calculated using "emmeans" package of R (https://doi.org/10.32614/CRAN.package.emmeans), if the authors use "lme4" package. The effect size on each visit will be calculated from this method.
4. The authors noted that they will use the Bonferroni correction for adjusting multiplicity in the moderator analyses. However, the family of statistical tests would not be meaningful one that the overall type-1 error rate should be rigorously controlled (not as like the multiple primary endpoints of confirmatory trials). I consider the multiplicity adjustment is not necessarily; if the authors favor the adjustments, the FDR controlling approaches would be more suitable (e.g., Benjamini-Hochberg method).
5. In the moderator analyses, the authors noted to report effect size information only for significant variables. However, the powers of three-way interaction tests might be insufficient and the effect size information itself might be more useful for readers. I suggest providing the information for all variables. Also, the outcomes for CBT and IPT groups will be calculable respectively, and their difference-in-difference outputs might be useful. The presentations adopted in Noma et al. (2019) might be useful.

Reference
Ishak KJ, Platt RW, Joseph L, Hanley JA, Caro JJ. Meta-analysis of longitudinal studies. Clinical Trials. 2007;4:525-39.
Noma H, Furukawa TA, Maruo K, et al. Exploratory analyses of effect modifiers in the antidepressant treatment of major depression: Individual-participant data meta-analysis of 2803 participants in seven placebo-controlled randomized trials. J Affect Disord. 2019;250:419-424.

VERSION 1 - AUTHOR RESPONSE

Reviewer: 1
Prof. Vladimir Trkulja, University of Zagreb School of Medicine
Comments to the Author:

This protocol describes a study intended to deal with an important question, and was submitted by a research group with a top-level expertise in the field (CBT, IPT) and the

proposed methodology (IPD meta-analysis). I have only a few comments that the authors might wish to consider.

Reply: We sincerely thank you for your thorough and insightful review of our manuscript. We deeply appreciate the time and effort you invested in providing such detailed feedback and thoughtful rationale, which have been invaluable in refining and strengthening our study protocol. We have addressed each of your comments to enhance the clarity and rigor of our work. Below are our responses.

<u>Comment 7</u>

1. The protocol depicts a research intended to provide a comprehensive insight into the relative efficacy of CBT vs. IPT in major depression, with a particular emphasis on potential effect moderators – as an effort of identifying patient subsets in which one or the other technique would be preferable (the list of the patient-level moderators to be explored is provided in the Supplement – by the way, it seems that the word "onset" is misspelled in the Supplement as "onsite"). The authors intend to include RCTs in which CBT-IPT were applied without concomitant antidepressants. Hence, antidepressant use is not among the potential effect modifiers. In the Introduction, the authors provide a brief overview of previous meta-analyses that intended to detect moderators of the CBT vs. IPT relative effect. Among many, one previous meta-analysis is mentioned (as ref. 5) that, apparently, indicated that regarding the depression symptom severity at the end of treatment, CBT was superior to IPT but only in patients without concominant antidepressants. This would imply that the authors of the present protocol considered it not needed to again address antidepressant use as a moderator (since, apparently, this question might be considered resolved). However, the cited meta-analysis (ref. 5) is – in my view – methodologically problematic in that the conclusion about superiority of CBT over IPT in patients without pharmacological treatments was not based on RCTs with direct CBT vs. IPT comparisons. Rather, the authors considered trials with CBT and estimated post- vs. predifferences, and trials with IPT (and post- vs. pre-differences). For both, the estimated pooled effects (Hedges g) were high (around 1.5), but apparently somewhat higher for CBT than for IPT (resulting in a "statistically significant difference", i.e., P-value of 0.037 for the difference between these post- vs. pre- differences). I sincerely doubt that the cited meta-analysis should be viewed as an indicator of the moderating effect of "concurrent antidepressants", and particularly not as evidence to support superiority of CBT over IPT in "non- antidepressant patients". Hence, as the proposed protocol intends to be the most comprehensive one to date, it seems plausible that this potential moderator is considered, since both CBT and IPT are commonly used together with antidepressants. Some other moderators (e.g., personality disorder) were also addressed in previous meta-analyses (as indicated by the Protocol authors), yet, personality traits are one of the proposed (potential) moderators. Hence, I do not see why a potential moderator, highly likely to be seen in daily practice, is omitted from the planed analysis.

Reply: We appreciate this comment on participant antidepressant use as a potential moderator. We agree that the cited meta-analysis (ref. 5) is methodologically limited and does not provide a valid conclusion regarding the superiority of CBT over IPT among patients not using antidepressants. Therefore, we do not consider this issue to be resolved for this reason. The primary reason we did not list antidepressant use as a potential moderator is that, we expect some RCTs might have listed this as an exclusion criterion, resulting in insufficient sample size to examine this variable. Although more recent RCTs have started to allow

medication use but exclude recent medication changes, they might have not assessed the type of medication. However, we acknowledge the importance of antidepressant use as a moderator and have now added it as a potential moderator in Supplemental Table 2. We have also corrected the word "onsite" to "onset" in this Table. Thank you for noting this typo.

Furthermore, we now remark on the limitations of ref. 5 in the Introduction on page 7-8:

"Nevertheless, this study has methodological limitations, as it compared pre-post effect sizes for patients receiving CBT with pre-post effect sizes for patients receiving IPT, instead of including RCTs that directly compared CBT and IPT."

<u>Comment 8a</u>

2. One of the proposed sensitivity analyses is an analysis limited to studies with "low risk of bias". The authors state "*Second, to examine whether the risk of bias of the included studies impacts outcome, we will repeat the analysis in studies with low risk of bias.*" I am aware that it is a common practice to conduct meta-analyses across the strata of "study quality". However, it seems to me that this practice is not very logical: i) if the objective of a meta-analysis is to generate the most (at the moment) precise estimate possible; and ii) the most unbiased one – why would anyone include "serious concerns" or "some concerns" RCTs (as an overall judgment, e.g., in line with the RoB-2 tool) into a meta-analysis? I mean, "serious concerns" means that the trial estimate is highly likely biased (whether towards or away from the null); i.e., it means that the trial was likely estimating bias, not the treatment effect. Why would one want to include an estimate of bias into the estimate of the treatment effect? Another logical fallacy – at least in my opinion – is the view that by showing, for example, "numerically similar estimates in low- and high quality RCTs", one documents "robustness of the findings": i) the high quality and low quality trials estimate two different things, and their "overall pooled estimate" is meaningless; ii) numerical similarity of estimates in low quality and high quality trials does not mean that "trial quality does not change the treatment effect estimate", because in the former, this is not what is estimated. We do not know what would have been the treatment effect estimate in low-quality RCTs – had they been of high quality. Not to mention limitations of the quality scores (e.g., Greenland&O'Rourke Biometrics 2001; 2:463). Hence, in any meta-analysis intended to indeed provide an unbiased and precise estimate of a causal treatment effect RCTs that are viewed as problematic in terms of various biases, should most likely be omitted. Alternatively, the main analysis could be based only on RCTs deemed to be fairly unbiased, whereas supplemental data could address those RCTs that are judged as likely biased to some relevant extent. Interpretation of possible similarity or differences between two estimates is not straightforward. Eventually, one could explore effects across different domains that have been considered as sources of bias. For example, estimate in trials with low risk of treatment allocation bias and those with a high risk of such a bias – to indicate, potentially, how much this type of bias skewes the estimates, and so on.

Reply: We sincerely appreciate your thoughtful and detailed discussion on the inclusion of studies with varying risks of bias in meta-analyses. We acknowledge the concerns regarding potential biases in trials with "some concerns" or "serious concerns" and agree that these biases could influence treatment effect estimates. Indeed, this is a longstanding limitation of meta-analysis. However, as recommended in the Cochrane Handbook for Systematic Reviews of Interventions, we believe that a comprehensive meta-analysis should incorporate all

available evidence, while addressing potential bias through sensitivity analyses. Below is our rationale:

1. **Maximizing available evidence**. Excluding studies solely based on risk of bias assessments could systematically omit valuable data and introduce its own form of bias.
2. **RCTs are generally strong designs**. While lower-quality studies may introduce uncertainty, they are still randomized controlled trials, one of the most reliable research designs. They are relatively strong when compared to other designs and their "low quality" is relative. The classification of studies into low, some concerns, or high risk of bias is based on manual coding and is not a purely categorical variable. While it depends on specific domains assessed within the RoB-2 framework, it involves subjective judgment. As a result, treating risk of bias as a rigid criterion for inclusion/exclusion could lead to an arbitrary cutoff that may not fully capture the nuances of study quality. Conducting sensitivity analyses allows us to account for these uncertainties more systematically.
3. **Balancing bias and precision.** We recognize that studies at high risk of bias may systematically over- or underestimate treatment effects. However, excluding them outright assumes that all biases necessarily distort results in a predictable way, which is not always the case. Some low-quality RCT may over-estimate the effects while others may under-estimate the effects. The trade-off between including all available evidence and minimizing bias is a well-known methodological challenge in meta-analysis, and no approach is perfect.

To address these concerns systematically, we plan to conduct a sensitivity analysis restricted to studies with a low risk of bias. This approach allows us to examine whether the inclusion of studies with some or serious concerns about bias impacts the overall findings. If discrepancies arise, this provides valuable insight into how study quality may influence effect estimates. We added the following sentence to illustrate the benefits of this sensitivity analysis on page 19:

"This approach allows us to examine whether the inclusion of studies with some or serious concerns about bias impacts the overall findings."

In this way, we follow the recommendations specified in the Cochrane Handbook for Systematic Reviews of Interventions, incorporate all available evidence in our meta-analyses, while addressing potential bias through sensitivity analyses.

Comment 8b

A number of other sensitivity analyses are planned. Most of those, however, induce some questions/considerations: i) one sensitivity analysis aims to include only the RCTs with inperson delivered treatments (guided self-help are excluded). Depicting this as a "sensitivety analysis" implies that all the moderators would be evaluated not only in the entire dataset, but also specifically in those with in-person therapies. Are in-person and guided self-help the same intervention? If they are, why would one expect a moderating effect of some patient-level characteristic to differ between the in-patient RCTs and self-help RCTs; or, if the two are the same intervention – why should one consider the analysis in only in-person RCTs as a "sensitivity analysis"? On the other hand, if they are not – why would one want to generate a pooled estimate across different types of treatments: in-person CBT vs in-person IPT and selfhelp CBT vs self-help IPT? Would it not be more plausible to first consider the "delivery type" as a trial-level moderator? And, if it turns-out to indeed be a moderator of the CBT vs. IPT difference, then test

person-level moderators in these two subsets defined by the "delivery type" which renders CBT and IPT different treatments (ie., in-person CBT is different from guided selfhelp CBT, in-person IPT is different from guided self-help IPT). At least to me, these are somewhat unclear points; ii) one sensitivity analysis is intended to include only RCTs with a high treatment quality on all criteria. Does this mean that here in-person treatment delivery and selfhelp delivery would be again analyzed jointly? What if it turns out that moderating effects of person-level characteristics are different in "delivery type" subsets…is it not counterintutive to then consider them jointly again in this analysis? iii) the fourth and the fifth sensitivity analyses were considered in (a) trials with IPT and trials with "briefer IPT counseling"; and (b) trials implementing Beckian CBT and trials with other CBT procedures. This is a bit confusing: if one criterion is "type of IPT", then either one could be compared to the Beckian CBT and "other CBT". These seem to be rather heterogeneous subsets. Let's assume that for example "age" is a personlevel moderator of the Beckian CBT vs. briefer IPT difference, but not of any other difference (e.g., Beckian CBT vs. IPT, non-Beckian CBT vs. briefer IPT, or non-Beckian CBT vs. IPT). Is it not possible that then a true moderating effect would not be seen, even if it existed, because it would be "diluted" by the lack of the moderating effect in all other comparisons? Overall, all these mentioned points listed in the section "Sensitivity analyses" are a bit confusing, at least to me as a half-lay person. It seems to me that all these listed planned analyses denoted as "sensitivity analyses" actually better fit into the evaluation of study-level moderators. I am not aware of the total number of existing primary RCTs comparing CBT to IPT, but I assume that the number is likely considerable (otherwise, by simple logic, it would not be plausible for the authors to plan the proposed research). This enables the meta-regression approach. The protocol never mentions the term "meta-regression" (on individual patient data); but, in general, the depicted models including treatment*patient-level moderator interaction terms are meta-regression models, and could also include treatment*study level moderator interaction terms. Moreover, patient-level and study-level covariate interactions could be evaluated (although this may not be required for achieving the main purpose of the planned study). Also, it might be plausible to consider trials in post-partum depression separately from those in major depressive disorder.

Reply: Thank you for your thoughts concerning the sensitivity analyses. We want to clarify that we meant these to be related to the primary efficacy analyses only. As such, we were not planning to evaluate all moderators in these various subsets of studies. Indeed, these analyses are assessments of study-level moderators. We can see now how our phrasing in the original version of the manuscript was not clear in this regard and have adjusted it as follows (page 19):

"We will conduct seven analyses to examine potential study-level moderators of CBT vs IPT efficacy concerning depressive symptoms at post-treatment."

We appreciate the suggestion on considering trials in post-partum depression separately from those in major depressive disorder. We have added an analysis examining this study-level moderator on page 19:

"Sixth, to examine the impact of depression type, we will perform the analyses separately in the subgroup of studies focusing on MDD and the subgroup of studies on post-partum depression."

__**Comment 8c**__

As the sixth "sensitivity analysis" authors state: "*Finally, to adequately account for baseline values of the outcome measure and assist in the comparison of this study's findings to prior work, we will repeat the primary outcome efficacy analysis using the strategy based on the approach recommended by Twisk and colleagues, which has been utilized in previous depression treatment IPD meta-analyses.*" I belive that baseline depression score-adjusted analysis of the end-oftreatment depression scores has to be the main analysis, not the sensitivity analysis. As depicted in the Protocol, the primary outcome is "end-of-treatment" Beck II depression score (as measured or as transformed from other depression-quantifying instruments). Such an outcome has to be analyzed with adjustment for the baseline depression severity (Twisk et al. Contemp Clin Trials Commun 2018; 10:80-85; Fitzmaurice GM, Laird NM, Ware JH. Longitudinal data analysis. Wiley 2004). Next, the authors state that for the purpose of adjusting for baseline, the "method described by Twisk" is to be used. In another part of the Protocol, they denote the analysis model as a multilevel model with two random intercepts (patient; study), and fixed effects treatment, time, treatmet*time interaction. This introduces some confusion. The "Twisk model" actually means that for longitudinal continuous outcome, baseline value of the outcome is treated as an outcome, not a covariate (ie., outcome value at time 0), and this approach adjusts for the baseline value when "treatment" is not in the model, but treatment*time interaction is in the model. However, the primary outcome is a single-time point outcome – at the end of treatment, i.e., there is only one post-baseline value. Hence, the model might be a simple model without repeated measures: hence, there are fixed effects – treatment, and baseline disease severity; and random intercept for "trial" (subjects clustered in trials) (although, some argue that the more appropriate approach is to consider trials as strata – e.g., Riley, Tierney, Stewart. Individual participant data meta-analysis, Wiley 2021 – Chapter 6 on one-step procedure – however, I cannot judge the appropriatness of one or the other approach- statistical expertise is needed). Another model could include also treatment*covariate interaction. The model could of course also explore the random slope for any of the tested covariates, or for the treatment*covariate interaction (when testing the possible moderation). The question is – what would be the purpose of including also random slopes, except to improve the model fit/reduce residual heterogeneity. This might need to be clearly stated (although, for the analysis of the primary outcome, I do not really see an explicit need for such an outcome). Hence, there seems to be some confusion about the explicit mention of the "method described by Twisk" for the primary outcome analysis and the way in which the model is described. However, an expert in IPD should evaluate this point. But, whatever method is used – the primary analysis of the primary outcome should include adjustment for the baseline depression score. In fact, all analyses of all outcomes should include adjustment for the baseline value of the respective trait. Additional argument in this regard: different trials will differ with respect to the baseline scores; I assume that the CBT vs IPT trials are generally not too large, so quite some imbalances in baseline severity between CBT and IPT (in all aspects of psychopathology) should be expected in many of the primary trials.

Reply: We acknowledge the importance of adjusting for the baseline values of the outcome variables. We now propose to apply the method described by Twisk et al., 2018 (equation 2c) as our default model of analysis. We have removed the sixth sensitivity analysis and now explicitly introduce our analysis strategy in the data-analysis section on page 17:

"To facilitate comparison of this study's findings to previous IPD meta-analyses on depression treatment efficacy, we will adopt the data-analysis strategy applied in the prior studies.[21–23] More details of this strategy can be found in prior publications.[21–23] This strategy is based on the approach recommended by Twisk et al,[47] and is chosen because it adequately accounts for baseline values and effectively addresses missing data. Specifically, it allows participants with only baseline data but missing assessments at post-treatment and/or follow-up to remain included in the analyses without requiring multiple imputation.[48]"

<u>**Comment 9**</u>

3. It is also likely that in many primary RCTs there would be imbalances in a number of other patient-level characteristics – potential confounders. The authors plan to generate an overall estimate of the treatment effect (ie., CBT vs. IPT difference), among other goals. This estimate, I believe, should be generated not only with an account for the baseline disease severity, but likely with adjustment for other relevant covariates (selected based on the authors' subject knowledge). I assume that not many primary RCTs (if any at all) have applied stratified randomization – but if any has implemented it, then I believe that the model should include stratifying factors as covariates. Overall – it seems reasonable to assume that most of the primary studies are limited in size, thus limited in their ability to detect and adjust for imbablances in relevant confounders, and that most have not reported effect estimates adjusted for the baseline severity. IPD meta-analysis is an opportunity to rectify these shortcomings of the primary studies.

Reply: Thank you for this thoughtful suggestion. We will check the included RCTs for applying stratified randomization. If implemented, we will conduct a sensitivity analysis adding the stratifying variable(s) as covariate(s). We did not choose this as our main analysis strategy, because we think the stratifying variable(s) might not be available for all studies, so that it might not be possible to conduct this analysis including the full set of studies. We now describe this in the Methods section (page 19):

"Finally, we will conduct a sensitivity analysis adding the variable(s) used for stratified randomization in the primary studies as covariate(s) to the model."

<u>**Comment 10**</u>

4. As mentioned, it is reasonable to assume that most of the primary studies are comparably limited in size. However, it is reasonable to assume that the really small ones (e.g. with 10-20 patients per treatment arm) would indicate larger treatment effects (particularly if published) than the somewhat large ones (e.g., 50-60-80 patients per treatment arm). The protocol anticipates the funnel plot analysis, but it seems to me that the trial size is a reasonable studylevel characteristic that could be considered as an effect moderator. An overall estimate based on the combination of overestimates (from very small trials), and estimates that are likely to be closer to the truth (ie., from reasonably larger trials) might not be too informative. If there are indications of the "effect inflation" (small trial effect), the really informative estimate of the CBT vs. IPT differences should probably come exclusively from somewhat larger trials.

Reply: We acknowledge that the sample sizes of the included studies can be small. Of the 11 CBT-IPT RCTs for which IPD have been collected so far, sample sizes range from 40 to 200, with 3 trials including 26 participant or less per treatment arm. Indeed, we consider the funnel plot analysis an assessment of trial size as an effect moderator, since it assesses effect size as

a function of sample size. Should these analyses indicate a small study effect, we will take this into account when interpreting the study's findings.

<u>**Comment 11**</u>

5. It is planned to test a rather large number of potential patient-level moderators. The rationale is clear, and this is a valuable effort – in the sense of contribution to the informed choices for individual patients. Still, strictly speaking, the proposed separate models (for the primary outcome) for individual potential moderators – as much as they might be informative – are also quite limited in their reach. Let's assume that age, baseline disease severity, and educational status are found to be effect modifiers, each in a separate analysis. This cannot provide an answer about a preferred choice for a middle-aged, moderately depressed and highly educated patient. Of course, no single trial or IPD MA could answer that, hence the authors rightfully depict the moderator exploration as a hypothesis-generating effort – findings of which should be evaluated in future RCTs. Still, a question remains- what other adjustments should be included in the moderator-testing models? In my opinion, if the essential condition for a reasonabley accurate estimate is to adjust for the baseline depression score – then it should be included in all models evaluating effect modifiers.

Reply: We acknowledge the limitations concerning the moderator findings described. We agree and have now adjusted the Data-analysis section (see also Comment 8c above), so that in all models examining potential moderators a correction for baseline depression is applied (page 17).

<u>**Comment 12**</u>

6. The Protocol envisages that eventually a model would be fitted to the primary outcome with all "significant moderators" as indepedents to evaluate their independence. The authors might want to consider the following: i) will the model also include (by default) the baseline depression score? I believe that it should; ii) Let's assume that e.g., 4 patient-level effect modifiers are detected – what fixed effects will the model include: treatment, baseline depression, each of the 4 effect modifiers, and interaction terms of each of them with treatment? Or?; iii) As outlined, it could be understood that the model would actually evaluate effects of these variables on the outcome, but this does not seem reasonable, i.e., what would be the purpose? It is true that, commonly, the effect modifiers are also confounders, i.e., that they are both associated with the outcome (or "affect the outcome") and also modify the effect of the exposure of primary interest (in this case - form of psychotherapy) – but, an effect modifier needs not be also a confounder (see e.g. VanderWeele TJ. Explanation in causal inference, Oxford University Press 2015). Assume, for example, that one of the effect modifiers is found out not to be "independently associated with the outcome" – does this disqualify it from being an effect modifier? What is gained by such an analysis?; iv) Is the criterion of "significant modifier" (which, presumably, would be based on "statistical significance of the interaction term") the appropriate one? I mean, it could be that not all trials provide data on a particular effect modifier, hence the sample size is reduced, and power is reduced, and the "test fails" in the sense that the p-value is, say, P=0.200 – should it be "disqualified" although by all reasonable arguments, or as shown in some previous study, it is likely to indeed be an effect modifier; v) Should all potential confounders be indiscriminately included? Let's assume that the history of a previous

treatment (yes/no) is associated with or "causes" (at least in part) the level of depression at the trial baseline, and that it affects the outcome by affecting the baseline disease severity, which is a factor that qualifies a patient for the trial – that is, that baseline disease severity is a mediator of the effect of "previous treatment" – by adjusting for both, one would actually "block" the effect of "previous treatment" on the outcome: does one really need to condition on both?

Reply: Thank you for this thoughtful discussion of our proposed evaluation of the independence of the moderators.

i) This model will be an extension of the basic analysis model (see Comment 8c for more detail) and as such will adjust for baseline depression

ii) The model would include the fixed effects:
- Time
- Moderator 1
- Moderator 2
- Moderator 3
- Moderator 4
- Time*Treatment
- Time*Moderator 1
- Time*Moderator 2
- Time*Moderator 3
- Time*Moderator 4
- Time*Treatment*Moderator 1
- Time*Treatment*Moderator 2
- Time*Treatment*Moderator 3
- Time*Treatment*Moderator 4

iii) The primary aim of this analysis is to examine whether the moderator effects are independent from one another. For instance, in a previous study (see Wienicke, F. J., Beutel, M. E., Zwerenz, R., Brähler, E., Fonagy, P., Luyten, P., ... & Driessen, E. (2023). Efficacy and moderators of short-term psychodynamic psychotherapy for depression: A systematic review and meta-analysis of individual participant data. *Clinical psychology review*, *101*, 102269), we found both the length of the current depressive episode and age of depression onset moderating treatment outcome. However, when modeled these moderators simultaneously, only length of the current depressive episode remained a significant moderator of treatment outcome, indicating the moderating effect of age of onset was accounted for by episode duration.

iv) This is a valid point regarding the issue of Type II error related to small sample sizes. We now propose to report confidence intervals for each moderator effect. When these are wide, this can be an indication that the power was too small to find an effect. We have added the following in this regard to Methods section on page 18:
"P values will be calculated with Kenward-Roger's approximate F test and 95% confidence intervals will be calculated using bootstrapping with 10.000 simulations."

v) If baseline depression and previous treatment were both found to moderate outcome, we would be interested to see if these effects are independent from one another, or whether the moderating effect of one is actually accounted for by the other, like in the example above.

<u>Comment 13</u>

7. Several analyses are envisaged to evaluate meta-biases. Among others, the authors intend to perform also a two-stage IPD – estimates from RCTs with available IPD will be combined with summary data from other RCTs for which IPD are not available. I have no idea about the expected number of IPD-available and non-available studies, but I presume that the latter might be even more numerous than the former. The authors plan to pool individual IPD estimates and summary estimates by the random-effects method. This appears as a reasonable effort in order to improve the precision of the estimates. However, I am not certain that this would contribute to the accuracy of the estimates. The effect estimates based on IPD could (and should) be adjusted for the baseline scores, or potentially other covariates – resulting, likely, in improved "unbiasedness". It seems somewhat counterintuitive to merge them with summary-based estimates likely generated from RCTs without adjustment for (at least) baseline scores. Assuming that there is a considerable number of IPD-available RCTs, the precision of the estimates would likely be considerable. It might not be the best choice to "taint" them with summary data estimates for the purpose of improving already satisfactory precision. The authors might consider a possibility of analyzing "summary data RCTs" separately, using for example bivariate meta-regression (availabe in metafor, based on van Houwelingen, Arends, Stijnen. Stat Med 2002; 21:589) – it could generate average outcome scores adjusted for average baseline scores by treatment. Then, in the next step, the overall single-stage IPD estimate and the summary data adjusted estimate might be presented to illustrate consistency (or, possible inconsistency) of the estimates.

Reply: Thank you for this thoughtful suggestion. So far, we have identified 14 studies meeting our eligibility criteria, with IPD being available for 11 studies. As such, we do not expect the number of IPD non-available studies being larger than the number of IPD-available studies.

Please note that the aim of this analysis is not so much to improve the precision of the estimates, but to assess potential data-availability bias. In other words, we aim to examine if there are systematic differences between studies for which IPD are and are not available. It is indeed important to make sure that effect sizes are calculated in a similar way to allow for a valid comparison. We appreciate the suggestion to adjust for baseline scores in the summary data RCTs, but expect that the requisite data needed to do so, might not be reported in all studies. Instead, we now propose to calculate effect sizes based on the data reported in the publications for all studies. Although this might result in less precise estimates for IPD-available studies, it will facilitate similarity in the comparison between studies for which IPD are and are not available and as such a more valid assessment of potential data-availability bias. This method has also been applied in prior IPD meta-analyses of depression treatments and we now describe this as follows in the manuscript (page 20):

"Additionally, we will conduct conventional meta-analysis 'subgroup analyses'– applying a fully random-effects analysis and pooling study-to-study variance across subgroups – to compare effect sizes between studies for which IPD were and were not available. For these analyses, Cohen's d effect sizes will be calculated based on the effect size data extracted from the publications."

<u>Comment 14</u>

8. The authors state the use of the method "described by Twisk" (Twisk et al. 2018) to analyze longitudinal data (e.g., data on outcome values at the end of treatment and later follow-ups), using an argument that this enables inclusion also of patients with only baseline scores, but missing later or follow-up data. This is a bit confusing. In the cited reference by Twisk and colleagues, 4 methods are described to analyze repeated measures data in RCTs, two of which are considered inadequate, since they do not account for the baseline values; and two that do. One of them, is the "longitudinal data analysis" – baseline value is considered as outcome, not covariate, and if the model does not include the factor "treatment" but only treatment*time interaction, then it provides adjustment of the post-baseline scores for the baseline value. An equally viable alternative is to use baseline as a covariate. But, inclusion of patients with no postbaseline values is meaningless – unless one implements some form of data imputation. Clearly, most of the IPD-available trials will have patients with missing data on the primary outcome (end-of-study depression score), or at later time-points, or missing data on other outcomes – is data imputation envisaged? There is no mention in the protocol, but the statement that the analysis will be on the ITT principles implies that it will. The type of data imputation needs to be declared. LOCF? Sensitivity analysis might actually be an analysis of data with a different type of data imputation (e.g., some of the multiple imputation methods).

Reply: We appreciate this comment regarding longitudinal data and missing values. We acknowledge the importance of clarifying our analytical strategy, particularly in relation to imputation and the inclusion of patients with missing post-baseline data. As described in response to Comment 8c above, we now propose to apply the method described by Twisk et al., 2018 (equation 2c) as our default model of analysis. This model allows participants with only a baseline value but missing post-treatment and/or follow-up assessments to remain included in the analyses.

This approach appropriately handles missing data under the assumption of missing at random (MAR) by utilizing all available data without the need for imputation. Twisk compared the results of mixed-model analyses with and without multiple imputation and found that it is not necessary to handle missing data using multiple imputations before performing a mixed-model analysis with this model (See Twisk, J., de Boer, M., de Vente, W., & Heymans, M. (2013). Multiple imputation of missing values was not necessary before performing a longitudinal mixed-model analysis. *Journal of Clinical Epidemiology*, *66*(9), 1022-1028.).

We have added the following sentence on page 17 to clarify our approach regarding missing values:
"To facilitate comparison of this study's findings to previous IPD meta-analyses on depression treatment efficacy, we will adopt the data-analysis strategy applied in the prior studies.[21–23] More details of this strategy can be found in prior publications.[21–23] This strategy is based on the approach recommended by Twisk et al,[47] and is chosen because it adequately accounts for baseline values and effectively addresses missing data. Specifically, it allows participants with only baseline data but missing assessments at post-treatment and/or follow-up to remain included in the analyses without requiring multiple imputation.[48]"

<u>Comment 15</u>

9. Clearly, if any method indicates lower end-of-treatment Beck II depression score, this would indicate "less depression" with the respective treatment. But this does not necessarily mean that either method actually relieved depression – it could simply mean

that with one vs. the other, patients were "less worse" than at the start of treatment. Of course, this is not likely, since both CBT and IPT are known to relieve depression. Still, one analysis intended to define MCID for Beck II based on data from 3 RCTs in patients with depression (Button et al. Psychol Med 2015; 40:3269), indicated, for example, average reductions in Beck II scores of -7 to -8 score points in patients who on the Global impression-improvement reported "feeling the same". With a large number of data in IPD, a difference between, e.g, by 7.5 vs. by 6.8 reduced scores (hence, assuming closely similar baseline – by 0.7 score difference in end-of-treatment score) could be found "statistically significant" – but it would be at the same time clinically meaningless. Moreover, the MCID with Beck II was found to depend on the baseline severity. In my opinion, to make the propsed analyses fully meaningful and practically relevant: i) all outcome score analyses need to be adjusted for the baseline value; ii) it would be informative to include another outcome (as it is done in e.g., regulatory RCTs of antidepressants) – proportion of people yielding at least 50% reduction in Beck II scores at the end of treatment. Alternatively, the outcome could be proportion of people experiencing MCID in Beck II score (which, to my understanding, could be approximated at around 25% reduction; Button et al. 2015; Kounali et al. Psychol Med 2022; 52:1875; Bdi Beck Depression Inventory Scoring - Consensus Academic Search Engine).

Reply: We appreciate the reviewer's insightful comment regarding the interpretation of changes in Beck Depression Inventory-II (BDI-II) scores and the importance of distinguishing statistical significance from clinical relevance. Concerning the first point, our primary analysis model now accounts for baseline differences (see Comment 8c above for more detail).

Following the second suggestion, we will include the observed proportions of participants yielding at least 50% reduction in BDI-II scores at the end of treatment as an outcome in our analysis. We have added the following description in the data-analysis section in this regard:

"We will also report observed response rates, defined as the proportion of patients achieving ⩾50% symptom reduction from pre- to post-treatment on the primary continuous depression measure."

We decided not to use the proportion of people achieving MCID because some studies did not use the BDI-II. Even though we will use existing transformation schemes to transfer other depressive measures scores into BDI-II, the derived MCID values may not accurately reflect clinically meaningful change across different measures, which could impact the validity of MCID. Therefore, we have opted to use the 50% symptom reduction threshold, which is a widely accepted and generalizable criterion for treatment response across different depression measures.

Reviewer: 2
Dr. Anne Doherty, Galway University Hospitals, National University of Ireland - Galway
Comments to the Author:

<u>**Comment 16**</u>
This is a well-written protocol which outlines a planned, systematic review in detail. The rationale and the background literature are well described. The design is thoughtful in

than rather than performing a meta-analysis of study outcomes, it will seek the original trial data in order to perform analysis at an individual level. The methods are appropriate to the stated aims and are described in detail.

Reply: We really appreciate your positive feedback on our study protocol. Your recognition of the rigor in our design, the clarity of our rationale, and the appropriateness of our methods is truly encouraging. We are particularly grateful for your acknowledgment of the value of conducting an individual participant data meta-analysis, as we believe this approach will provide deeper insights beyond what traditional meta-analyses can achieve. Thank you for your time and consideration in reviewing our work.

Reviewer: 3
Dr. Tanya Hauck, Centre for Addiction and Mental Health
Comments to the Author:
Overall this is a very detailed and thoughtful protocol.
The authors will use participant level data from randomised clinical trials to compare IPT and CBT for depression. The goal of the study is to conduct an individual participant data meta-analysis to understand treatment efficacy and how baseline characteristics moderate outcomes.
It is well described and detailed, and the methods are reasonable.
I have a few minor points to consider, and understand that it may now be possible to address all of them, also due to data limitations:

<u>Comment 17</u>
(a) Another important domain of baseline characteristics may be therapist factors. For example, therapist training, therapist profession/role (psychologist, psychiatrist, social worker, peers, etc.), and the use of rating scales such as CTRS to rate whether the therapy is being performed properly. At least in my jurisdiction it is vastly easier to obtain training in CBT compared to IPT, and therapist familiarity with the model may also drive outcomes.

Reply: Thank you very much for your positive feedback. We most appreciate your time and effort reviewing our manuscript and providing these thoughtful suggestions.

We agree that therapist factors are important to consider. We, therefore, will examine CBT and IPT treatment quality with regard to therapist training, manual use, and therapy integrity ratings (including the CTRS). We describe this in the manuscript as follows (page 16):

"CBT/IPT treatment quality will be examined according to the following criteria: 1) whether a treatment manual was used, 2) whether the therapy was provided by trained therapists, and 3) whether treatment integrity was verified.[22]"

We will examine the potential influence of these therapist factors by repeating the primary efficacy analysis in studies that score high on these criteria (see manuscript page 19).

In addition, we have planned a separate project examining therapist effects, including an assessment of therapists' profession and roles, in more detail. The protocol for this project is described elsewhere (https://osf.io/bcrn7).

<u>Comment 18</u>

(b) IPT is formally meant to focus on four general areas (grief, conflict, changes in relationships/life, social isolation) and it is possible someone may be appropriate for CBT who does not technically qualify for IPT. If it is possible to see this eligibility criteria in the data, it would help to include it.

Reply: Thank you for this insightful thought. Although presence or absence of specific IPT problem areas and appropriateness for IPT would be interesting moderators to consider, identification of the focal IPT problem area is part of the IPT treatment process, typically occurring at the end of the second or third therapy session. The IPT problem area is not defined by the presence or absence of life events; rather it is selected collaboratively with the patient and giving strong consideration to the temporal link between interpersonal issues and mood symptom onset. There would be no comparable data available for CBT participants because by definition therapists do not undertake the therapeutic work needed to identify and operationalize the IPT problem area. Therefore, unfortunately, we will be unable to consider this issue in our planned analyses.

<u>Comment 19</u>

(c) On page 17 (page 10) it states that cognitive restructuring defines CBT. I would argue that behavioural activation is equally effective and part of the wider CBT framework, and is in the CANMAT 2016 guidelines (2016, Vol. 61(9) 524-539, Can J Psych) as equivalent to CBT for acute phase depression. I am not sure why BA is excluded? It may be particularly effective in some populations. If there are clear reasons for excluding BA, please detail them if possible.

Reply: We agree that BA often is part of CBT and we have now rephrased our description of the CBT eligibility criteria in hopes of more accurately reflecting this (page 10):

"To be determined as CBT, an intervention must be a manualised psychotherapy in which cognitive restructuring is the main treatment component, <u>typically also incorporating some behavioral techniques as well, such as activity scheduling</u>. Beck et al.'s model is considered to be the prototype of CBT,[26] but other CBT manuals can also be eligible for the current study.[22,24]"

We also agree that BA as a stand-alone treatment is equivalent to CBT in terms of efficacy. Our literature searches (up to date to May 1st, 2024), however, did not identify any RCT comparing BA and IPT. For this reason, and to reduce potential heterogeneity in the treatments examined, we decided to focus on the comparison of CBT and IPT in this project.

We do agree that BA may be particularly effective in some populations and identifying the patient characteristics defining these populations is an explicit aim of one of our other projects (https://pubmed.ncbi.nlm.nih.gov/35946068/).

<u>Comment 20</u>

(d) Finally, it may be worth adding to the limitations that in real practice, most jurisdictions (in public or insured systems, versus private-pay), the vast majority of clients worldwide (even in wealthy nations) will never have the option of selecting IPT versus CBT, and that resource limitations and availability of any evidenced based psychotherapy is a major concern.

Reply: Thank you for this suggestion; this is a great point. We have added it as a limitation in the Discussion section (page 22):

“Finally, in real-world settings, patients might not have the chance to choose between IPT and CBT (as well as other evidenced-based psychotherapies) due to limited resources and constrained treatment accessibility. While our findings will provide valuable insights into the comparative efficacy of these treatments, their applicability will depend on healthcare access and policy considerations.”

Reviewer: 4
Prof. Hisashi Noma, The Institute of Statistical Mathematics, The Graduate University for Advanced Studies
Comments to the Author:
This paper provides a protocol for an individual participant data meta-analysis that compares the efficacy of cognitive behavioral therapy and interpersonal psychotherapy. This study also aimed to explore their effect moderators. The research methods are well considered and well presented. I’d like to provide several minor comments and suggestions.

Comment 21
1. For calculating I^2-statistics, how are they defined in the one-stage analyses by mixed-effects models?

Reply: Thank you for your time and effort reviewing this manuscript. We value your thoughtful feedback focusing on the proposed methods of our study.

We can see how the manuscript would benefit from a more detailed explanation for calculating the I^2-statistic and have added the following to the relevant Methods section (page 17):

“We will perform one-stage IPD meta-analyses on intention-to-treat samples, as far as data are available, using three-level mixed-effects models (level 1: measurement moment; level 2: participant; level 3: study) with restricted maximum likelihood estimation.[21–23] As some participants may seek additional help during the follow-up period, which usually cannot be controlled, follow-up data will be excluded from post-treatment analyses. To examine heterogeneity, the variance among studies as a proportion of the total variance (i.e., I^2-statistic) will be calculated by dividing the random effect variance for study by the sum of all variance components. Furthermore, the normality assumption will be checked by visually inspecting histograms of (standardized) residuals.[22]”

Comment 22
2. P.17, line 35: “The models will contain random and fixed slopes for time, treatment, and time × treatment interaction on the study level.”
Does this statement mean all of these coefficients are random? Or, some of them are not random? In the statistical analysis section, they should be explicitly notified. Ishak et al. (2007) will be helpful for the modelling strategies.

Reply: Thank you for the helpful literature suggestion. We can see how this model description in the original version of the manuscript was unclear. Following the recommendations specified in Ishak et al. (2007), we have now rewritten it to clearly indicate that all coefficients are random (page 18).

Comment 23

3. P.17, line 38: "The regression coefficient of the time × treatment interaction will be considered to reflect the treatment effect."
The coefficients of interaction terms would be difficult to be interpreted. The least-squares means can be easily calculated using "emmeans" package of R (https://doi.org/10.32614/CRAN.package.emmeans), if the authors use "lme4" package. The effect size on each visit will be calculated from this method.

Reply: This is very helpful, thank you. We indeed aim to use the R "lme4" package to compute least-square means for models in which coefficients that cannot be easily interpreted.

Comment 24

4. The authors noted that they will use the Bonferroni correction for adjusting multiplicity in the moderator analyses. However, the family of statistical tests would not be meaningful one that the overall type-1 error rate should be rigorously controlled (not as like the multiple primary endpoints of confirmatory trials). I consider the multiplicity adjustment is not necessarily; if the authors favor the adjustments, the FDR controlling approaches would be more suitable (e.g., Benjamini-Hochberg method).

Reply: We agree. Following this suggestion, we will not apply an adjustment for multiplicity and have removed the relevant section on Bonferroni correction from the Methods.

Comment 25

5. In the moderator analyses, the authors noted to report effect size information only for significant variables. However, the powers of three-way interaction tests might be insufficient and the effect size information itself might be more useful for readers. I suggest providing the information for all variables. Also, the outcomes for CBT and IPT groups will be calculable respectively, and their difference-in-difference outputs might be useful. The presentations adopted in Noma et al. (2019) might be useful.

Reply: Thank you for this suggestion. We agree that it is important to report effect size information for all moderators examined and have adjusted the relevant section (page 18) as follows:

"For categorical moderator variables, separate treatment effects will be estimated for each moderator category."
